# Modulation of the Earliest Component of the Human VEP by Spatial Attention: An Investigation of Task Demands

**DOI:** 10.1093/texcom/tgaa045

**Published:** 2020-08-05

**Authors:** Kieran S Mohr, Niamh Carr, Rachel Georgel, Simon P Kelly

**Affiliations:** Cognitive Neural Systems Lab, School of Electrical and Electronic Engineering and UCD Centre for Biomedical Engineering, University College Dublin, Dublin 4, Ireland; Cognitive Neural Systems Lab, School of Electrical and Electronic Engineering and UCD Centre for Biomedical Engineering, University College Dublin, Dublin 4, Ireland; Cognitive Neural Systems Lab, School of Electrical and Electronic Engineering and UCD Centre for Biomedical Engineering, University College Dublin, Dublin 4, Ireland; Cognitive Neural Systems Lab, School of Electrical and Electronic Engineering and UCD Centre for Biomedical Engineering, University College Dublin, Dublin 4, Ireland

**Keywords:** C1, spatial attention, V1, visual, visual evoked potential

## Abstract

Spatial attention modulations of initial afferent activity in area V1, indexed by the first component “C1” of the human visual evoked potential, are rarely found. It has thus been suggested that early modulation is induced only by special task conditions, but what these conditions are remains unknown. Recent failed replications—findings of no C1 modulation using a certain task that had previously produced robust modulations—present a strong basis for examining this question. We ran 3 experiments, the first to more exactly replicate the stimulus and behavioral conditions of the original task, and the second and third to manipulate 2 key factors that differed in the failed replication studies: the provision of informative performance feedback, and the degree to which the probed stimulus features matched those facilitating target perception. Although there was an overall significant C1 modulation of 11%, individually, only experiments 1 and 2 showed reliable effects, underlining that the modulations do occur but not consistently. Better feedback induced greater P1, but not C1, modulations. Target-probe feature matching had an inconsistent influence on modulation patterns, with behavioral performance differences and signal-overlap analyses suggesting interference from extrastriate modulations as a potential cause.

## Introduction

The ability to use attention to enhance perceptual representation in line with current goals is one of the hallmark features of higher brain function. Spatial attention—the enhancement of perception at certain relevant locations in space—has been studied particularly intensively over decades of psychophysical and human and animal neurophysiology research (Posner 1980; Hillyard et al. 1998; Gilbert and Sigman 2007; Carrasco 2011; Posner 2016). A pervasive debate has centered on whether attention modulates neural activity early or late in the visual processing hierarchy. Animal neurophysiology has provided ample evidence that spatial attention can enhance visual evoked neural responses of spatially tuned neurons in the primary visual cortex (V1) (Vidyasagar 1998; McAdams and Maunsell 1999; McAdams and Reid 2005; Li et al. 2006; Briggs et al. 2013), though with significant variations depending on task-demand factors (Chen et al. 2008; Briggs et al. 2013) and stimulus contextual factors (Motter 1993; Luck et al. 1997; Ito and Gilbert 1999; Gilbert and Sigman 2007). There is also ample human funtional magnetic resonance (fMRI) evidence that spatial attention modulates blood oxygen level-dependent activity in V1 (Martínez et al. 1999, 2001). However, whether V1 activity is modulated specifically during the initial feedforward sweep of visual processing has remained unclear and controversial, partly because human fMRI lacks the temporal resolution to distinguish initial afferent from longer latency reentrant or preparatory activity. Electroencephalography (EEG) and magnetoencephalography (MEG), which have much higher temporal resolution, have been used to measure initial afferent V1 activity via the first, “C1” component (~70–90 ms poststimulus) of the visual evoked potential (VEP), which exhibits variations in topography across stimulus locations that are uniquely consistent with the geometry of human V1 (Jeffreys and Axford 1972; Clark et al. 1994; Di Sereno et al. 2002; Kelly, Schroeder, et al. 2013; Kelly, Vanegas, et al. 2013). Investigations of spatial attention modulation of the human C1 have yielded mixed results, with some reporting modulations (Proverbio et al. 2007; Kelly et al. 2008; Poghosyan and Ioannides 2008; Fu et al. 2010b; Rauss et al. 2012; Dassanayake et al. 2016) but most failing to find a modulation (Heinze et al. 1994; Johannes et al. 1995; Mangun 1995; Clark and Hillyard 1996; Bruin et al. 1998; Hopfinger and Mangun 1998; Lange et al. 1998; Martínez et al. 1999; Di Russo et al. 2003; Fu et al. 2005; Schuller and Rossion 2005; Hopfinger and West 2006; Yoshor et al. 2007; Di Russo et al. 2012; Ding et al. 2014). This is in spite of the fact that these studies routinely find modulations of later components such as the P1 (~100–140 ms poststimulus) and N1 (~140–160 ms poststimulus). The inconsistency of C1 modulations recently became yet more puzzling when 2 studies conducted rigorous, well-powered tests of C1 modulation using the same spatially cued target detection task as was used in a previous study reporting a robust modulation (Kelly et al. 2008), yet did not replicate the effect (Baumgartner et al. 2018; Alilović et al. 2019; see also Mohr and Kelly 2018; Slotnick 2018). In the original study by Kelly et al. (2008), leftward or rightward tilted gratings appeared in every trial and participants monitored these for the presence of rare, superimposed targets, which were faint rings whose arc width was comparable with the spatial frequency of the gratings. As we recently pointed out (Kelly and Mohr 2018), there were subtle differences between the original experiment of Kelly et al. (2008) and the replication experiments of Baumgartner et al. (2018) and Alilović et al. (2019) that may explain the discrepancy, opening up a valuable opportunity to gain new insights into the principles of operation of spatial attention, and most importantly, how modulation patterns are tailored to specific task demands. We detail 2 such discrepancies below.

One difference was the level of performance feedback provided to participants. In all cases, task difficulty was adjusted online based on participant behavior by moving through 11 different difficulty levels (determined by the extent of the luminance reduction of the ring). However, in the replication experiments, participants did not know their current difficulty level and were simply given their hit, miss and false alarm rates at the end of each block. By contrast, in the original experiment, participants were additionally told their difficulty level and were encouraged to try to make it to the hardest level. This difference could be vital as the participant’s knowledge of their attained difficulty level may have instilled motivation to use potentially costly attentional resources (Warm et al. 2008) in order to maximize their achieved difficulty level. By contrast, the provision of hit, miss and false alarm rates as the only feedback may not strongly motivate subjects to make their best effort because the adaptive staircasing allows a given set of such rates to be achievable at any difficulty level.

Another discrepancy between the experiments was that the background in the original experiment was dark relative to the grating stimuli, and thus grating onset incurred a net increment in luminance at the location where the grating appeared. This meant that gratings contained both high- and low-spatial frequency content (Kelly and Mohr 2018). By contrast, both replications used pure-contrast gratings, which had a high-spatial frequency component only, and thus low-spatial frequencies were unique to the target stimuli where the superimposed ring induced a net luminance reduction. Thus, a viable strategy for task performance in the replication experiments would have been to focus on low spatial frequency-coding neurons (bypassing the neurons that code for the nontarget probe stimulus), a feature-selective strategy that may not be beneficial in the original experiment since low-spatial frequencies were not unique to the targets in that case. By this account, low-spatial frequency-tuned neurons in V1 may have been modulated in the experiments of Baumgartner et al. (2018) and Alilović et al. (2019) but not reflected in the measured C1 component because it probed only high spatial frequencies. This account is consistent with the feature similarity gain model (Treue and Trujillo 1999) and a more recent development of it whereby the strategy is to boost the most discriminatory neurons (Navalpakkam and Itti 2007). In general, feedback provision and target-probe feature similarity form central components to any task used to probe the operations of attention, yet their impact has never before been systematically examined in any human VEP study of spatial attention.

To examine the potential influence of these task factors, we conducted 3 experiments in which participants detected targets that were superimposed on gratings, similar to the study of Kelly et al. (2008). In Experiment 1, we sought to replicate the original experiment as closely as possible, albeit in a different lab. In the second and third experiments, we used a version of the task in which the superimposed target was a second orthogonal grating to enable more direct matching of stimulus features between the target and the nontarget stimulus used to probe attention effects. Experiment 2 employed 2 different feedback regimes reflecting the original and repeat experiments, respectively, while holding the target stimulus constant. We anticipated that a C1 modulation would be present when participants were given detailed performance feedback but that the modulation would be diminished or potentially absent when this feedback was not provided. In Experiment 3, we compared the Gabor-on-Gabor task condition with a condition in which the superimposed target was defined by a uniform luminance disk (dissimilar features to the probe stimulus), with feedback regime held constant at its high level. We hypothesized that when target and probe features were similar, attention would boost target and probe features alike and that a C1 modulation would be observed. By contrast, when target features were dissimilar to probe features, we anticipated that attention would instead focus on target features only and that a C1 modulation would therefore not be observed as it is probed by probe stimuli.

Finally, although the prevailing view is that the C1 is generated in area V1, this has not gone unchallenged, with suggestions that neighboring V2 and V3 contribute as well (Edwards and Drasdo 1987; Maier et al. 1987; Ales, Carney, et al. 2010; Ales, Yates, et al. 2010; Ales et al. 2013), highlighting the need to consider signal overlap when interpreting the C1 (Qu and Ding 2018). For example, since V2 and V3 oppose V1 anatomically for much of the visual field (and consequently in terms of scalp polarity) modulations of all 3 areas may tend to cancel out on the scalp, or at least become diminished to the point that they are difficult to detect. Another often cited source of signal overlap is the P1, which is difficult to distinguish from the C1 at lower visual field locations where they are alike in polarity and have similar topographies (Di Russo et al. 2003). Together, these considerations motivate efforts to attempt to mitigate signal overlap as much as possible. Thus, in all 3 experiments, we conducted analyses both with and without a current source density (CSD) transformation applied to the EEG data, which attempts to improve the spatial resolution of EEG by converting raw voltages to their second spatial derivative and thus isolating activity more locally situated on the scalp (Kayser and Tenke 2006). The comparison of CSD- and non-CSD transformed data may offer some glimpse into the impact of signal overlap on C1 dynamics that may not be available from either version of the data alone.

## Method

### Multifocal Mapping

The C1 component of the VEP is classically observed as a negative midline Occipital deflection for most upper visual field locations and a similar positive deflection for lower visual field locations. This characteristic feature of the C1 forms an important part of the cruciform model describing the changes in topography as a function of stimulus location that is uniquely consistent with V1 morphology (Jeffreys and Axford 1972). However, the precise folding pattern of the cortical surface along the Calcarine banks that house area V1 varies considerably between individuals (Rademacher et al. 1993; Amunts et al. 2000). Consequently, the visual field locations that yield the strongest projections onto the scalp vary accordingly from person to person. Thus, prior to each experiment we implemented a pattern pulse multifocal visual evoked potential (PPMVEP) paradigm to map early visual responses of individual participants in order to choose stimulus locations with strong early visual activity (James 2003; Vanegas et al. 2013). For this, we used a “dartboard” stimulus composed of a checkerboard annulus with an inner radius of 2.75^o^ and an outer radius of 7.25^o^. This stimulus was divided angularly into 32 wedges of 11.5^o^ polar angle, each containing a 2 × 4 check pattern (angle × eccentricity). The wedges followed independent rapid pulse onset presentation protocols that were governed by a 4095 frame m-sequence (Baseler et al. 1994). These are sequences of binary numbers that are orthogonal to time-lagged versions of themselves. Thus, from the original m-sequence we took an additional 31 time lags to produce 32 independent pulse streams for the 32 wedges. Two-frame stimulus pulses (26.6 ms) occurred at transitions from −1 (the “off digit”) to 1 (the “on digit”) in the m-sequence. To limit the overall pulse speed, we kept only every fourth pulse, as has been done elsewhere (Vanegas et al. 2013). This yielded a protocol with 256 stimulus pulses for each location extending across a time period of 54.6 s, which we repeated 10 times with different m-sequences yielding 2560 pulses per location. Note that the use of a PPMVEP mapping procedure replaces the probe session that was utilized in previous renditions of this experiment (Kelly et al. 2008; Baumgartner et al. 2018; Alilović et al. 2019). The PPMVEP was favored here due to its ability to cover a greater number of spatial locations and to yield a high signal to noise ratio in a much shorter timeframe than the VEP-based probe sessions used in the previous experiments (10 min protocol, carried out prior to the main experiment on the same day compared with a separate probe session of ~1 h in duration on a prior day).

To choose locations, we calculated average epochs for each wedge position and visualized the resulting scalp topographies (calculated from 12 ms windows shifted by 8 ms intervals from 50 to 90 ms). First, we rereferenced the data to the average of scalp channels and band-pass filtered the data between 1 and 45 Hz with a fourth-order Butterworth filter. Epochs were baseline-corrected to the average activity between −26 and 40 ms post stimulus onset and average epochs were calculated for each wedge position by taking the average of 100 bootstrap samples of epochs, each containing 300 epochs. Two stimulus locations were chosen by the experimenter through visual inspection of the topography, employing the following criteria: (1) The locations included an upper field location and a lower field location at an angular distance of no less than 120^o^, chosen to strike a balance between the flexibility needed to find optimal locations on the one hand and mitigation of the risk that a single locus of attention could be spread between 2 nearby locations on the other (Müller et al. 2003). (2) The topography showed a strong negative polarity between 70 and 90 ms for the upper field location and a strong positive polarity for the lower field location. (3) Where possible, preference was given to locations with a midline or near-midline topography; this was because although lateral topographies are consistent with V1 anatomy both near the vertical meridians and in scenarios where the Calcarine sulcus is not perfectly horizontal, such lateral topographies are more likely to overlap with the P1 component of the VEP (Di Russo et al. 2002). The chosen locations for each participant are displayed for all 3 experiments in Supplementary Figure 1.

### Experiment 1—Ring Experiment (Reproduction of Original)

#### Participants

Sixteen healthy young adults took part in this experiment (7 females, 13 right handed) with a mean age of 23.2 years (SD = 2.5). They were compensated for their participation with a lump sum of €30. All participants gave written informed consent, were over the age of 18, had normal or corrected to normal vision and reported no neurological or psychiatric conditions. All operations were approved by the Human Research Ethics for Sciences board of University College Dublin and adhered to the guidelines set out in the Declaration of Helsinki. One participant who did not exhibit a clear C1 signal was excluded from C1 analyses (leaving a sample of *N* = 15) but was retained for other analyses.

#### Stimuli

**Figure 1:**
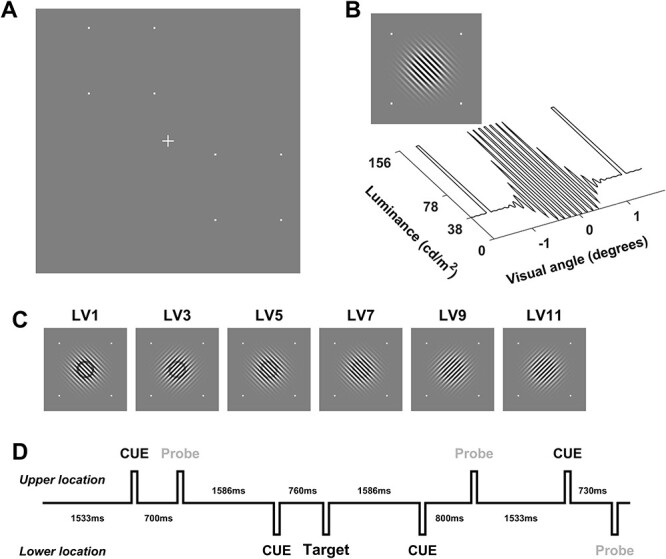
Stimuli used in experiment 1. (*A*) Screen layout with 2 example stimulus locations marked by the 8 white dots (*B*) A probe (top) and a diagonal slice of this image (bottom) showing the net luminance increment. (*C*) Odd difficulty levels. (*D*) An example of 4 consecutive trials showing the trial by trial cue and the random inter stimulus intervals.

Stimuli were identical to those used in the original experiment of Kelly et al. (2008), even using the same bitmap files (albeit on a different monitor). They were presented in a dark, sound-attenuated chamber on a 1024 × 768 Dell E771p CRT monitor (32.5 × 24.5 cm) at a distance of 125 cm from the participant who was seated upright without a chin rest in order to mimic the original setup. The stimuli included a probe stimulus, a target stimulus, a fixation cross, and white squares to demarcate the corners of the regions of space in which probes and targets appeared (see Fig. 1*A*). The latter were square regions of 2.75^o^ diameter centered at an eccentricity of 4^o^ from fixation at polar angles indicated by the PPMVEP mapping. The squares demarcating this region were 5 × 5 pixels each (~0.07^o^ × 0.07^o^). The probe stimulus was a full contrast saturated Gabor stimulus presented on a gray background of luminance 38.4 cd/m^2^. These original stimuli were constructed using the 0–255 brightness scale without gamma-correction, and therefore the middle brightness of 127 would have corresponded to a physical luminance significantly below the midpoint between the white (255) and black (0) luminance levels, hence creating the net luminance component in the stimuli. Since we had no record of the physical luminance of the monitor used in the original experiment, we had to assume that the typical luminance and gamma-curve properties of the monitor used here are representative of those of the original (also a CRT monitor). Importantly, the stimulus properties in this experiment differ from those of the failed C1 modulation replications (Baumgartner et al. 2018; Alilović et al. 2019) in the same qualitative ways as the original experiment (the replication experiments did apply gamma-correction and probe stimuli thereby had no net luminance component). The Gabors were oriented at 45^o^ or 135^o^ (randomized on each trial) with a spatial frequency of 5.89 cycles per degree, a sigma value of 0.648^o^, and truncated such that its full diameter was 4^o^. Saturation was achieved by defining the Gabor on [−1, 1] space, adding 0.5 to bring this to [−0.5, 1.5] space, and then enforcing a maximum and minimum of 1 and 0, respectively. The net luminance increase was achieved by linearly translating this [0, 1] space to the brightness levels of the monitor, which followed a gamma function with respect to luminance such that the middle brightness level corresponded to one quarter of maximum luminance. Thus, the resulting Gabor stimulus was asymmetrical such that the brightest segments had approximately 4 times the luminance of the background with the dark segments having close to 0 luminance (see Fig. 1*B*). Targets were rings of luminance reduction of the above Gabor stimulus with an outer radius of 0.45^o^ and an inner radius of 0.4^o^. These rings were introduced by multiplying the Gabor stimulus, prior to saturation, by a fraction within the confines of the ring. This fraction varied among 11 equally spaced values between 0.4 and 0.9, which defined 11 different difficulty levels of the target (see Fig. 1*C*). Note that since these rings were calculated prior to saturation, the rings grew darker with increasing eccentricity in accordance with the Gaussian profile, but the reductions were also offset somewhat by the saturation itself. Thus, at the outer edge where reductions were greatest, the corrected fractions of maximum brightness level were equally spaced between 0.424 and 0.954. At the inner edge, where reductions were smallest, the corrected fractions were equally spaced between 0.498 and 1.122 (note that since the last 2 of these numbers are above 1, these rings are in fact clipped, becoming slightly narrower). Finally, the fixation cross was a 0.44^o^ × 0.44^o^ vertical white cross with an arm width of 0.04^o^.

#### Procedure

Prior to commencement of the experimental session, participants completed the PPMVEP mapping protocol described above and 2 spatial locations were chosen for use in the experimental task that yielded strong early visual responses. The experimental task was a spatially cued target detection task. Participants maintained fixation on the central cross and were cued on a trial by trial basis to covertly attend to one of the 2 chosen locations by means of a white arrow that appeared between 700 and 800 ms prior to stimulus onset (uniformly distributed) for a duration of 100 ms. The cue-stimulus interval was jittered in this way to dampen the impact of cue-induced alpha phase-reset on the stimulus evoked response through phase cancelation. The stimulus then appeared (also for 100 ms) and participants responded by mouse click to targets at the attended location, while ignoring all stimuli at the uncued location. A randomly selected interval of either 1533 or 1586 ms (see Fig. 1*D*) preceded the cue for the subsequent trial, again with the 50 ms separation designed to dampen the impact of alpha phase-reset. Difficulty fluctuated online among 11 levels based on the participant’s performance. Difficulty increased by one level following 2 correct detections and decreased by one level following 2 false alarms or a single miss. (All counters were reset to zero upon a level change so a single false alarm or a single hit would be negated if the level changed; see Supplementary Fig. 2.) Participants were given feedback upon occurrence of all of these events by means of one of 4 auditory tones. A 150-ms tone at either 800 or 400 Hz indicated either the first hit or the first false alarm, respectively; a seamless pair of 100-ms tones (800–1066.66 Hz) indicated a level up (along with a text print of the new level); and similarly for a level down (400–266.66 Hz). Between blocks, the difficulty level was adjusted by taking the average level of the second half of the preceding block −2 in order to facilitate an upward progression in every block. Each trial had a 17.5% probability of containing a target, which appeared at the attended location only. Of the remaining 82.5% of trials, probes appeared at either location with equal probability. To ensure that comparisons between the attention conditions were made based on identical stimulation conditions, trials in which targets appeared were not included in analyses. Blocks comprising 102 trials were divided into quarters with short breaks between each one. At the end of each block, participants were shown a pie chart of their misses and false alarms on that block as well as a trial × difficulty level line plot depicting their level progression since the beginning of the experiment (see Supplementary Fig. 3 for an example of such a graphical display). After a practice block (or more if the participant requested), participants completed 10 such blocks totaling 1020 trials, of which 840 were probes, yielding 210 probes for each location and attention condition.

### Experiment 2—Feedback Experiment

#### Participants

Seventeen healthy young adults took part in this experiment (10 Females, 14 right handed) with a mean age of 22.1 years (SD = 5.8). They were compensated for their participation with either a lump sum of €20 or with research participation credit. Two participants completed only one condition of the experiment (see “Procedure” below), leaving 15 participants who completed the full experiment (9 females, 13 right handed; mean age = 21.4, SD = 4.8). All participants gave written informed consent, were over the age of 18, had normal or corrected to normal vision and reported no neurological or psychiatric conditions. All operations were approved by the Human Research Ethics for Sciences board of University College Dublin and adhered to the guidelines set out in the Declaration of Helsinki.

#### Stimuli

**Figure 2:**
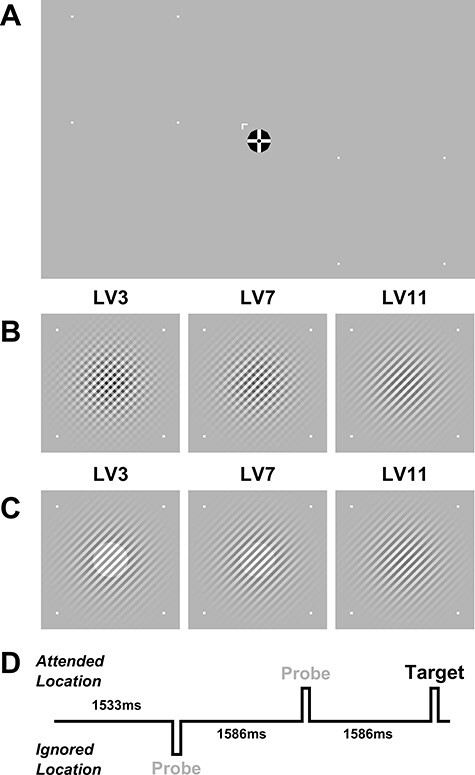
Stimuli used in experiments 2 and 3. (*A*) Screen layout with 2 example stimulus locations each marked by 4 white dots. The arrow cue was displayed at the start of the block and following the 3 short breaks but was not present throughout the trials. The fixation cross and location markers however were always present. (*B* and *C*) The similar-features target (orthogonal Gabor) and dissimilar-features target (luminance disk) shown at 3 different difficulty levels. (*D*) Example timeline of 3 consecutive trials showing the random inter stimulus interval.

Stimuli were again presented on a 1024 × 768 CRT monitor (32.5 × 24.5 cm) in a dark, sound attenuated chamber but this time at a distance of 57 cm from the participant who was seated at a chin rest. As before, stimuli included a probe and target stimulus, a fixation object, and location markers (white squares of ~0.1^o^ × 0.1^o^). The stimulus region was wider in this experiment (4.5^o^ diameter) and presented at greater eccentricity (6^o^), with precise locations again dictated by PPMVEP mapping. The probe stimulus remained a Gabor, but this time with no net luminance component and it was displayed at 60% contrast against a gray background of luminance 52 cd/m^2^. As before it was oriented at 45^o^ or 135^o^ randomly but its spatial frequency was slightly lower (4 cycles per degree) and it spanned a larger area (sigma value of 1.5^o^, truncated such that its full diameter was 6^o^). A different target stimulus was chosen in this experiment (and experiment 3) in order to facilitate more direct matching of stimulus features while also enabling us to explore whether the original result was specific to a ring-detection task. This target was a second Gabor stimulus (superimposed onto the probe by arithmetic addition), which was identical to the probe but oriented orthogonally. Importantly, since the orientation of the probe was random, so too was the orientation of the target, so participants could not carry out the task by focusing selectively on either orientation. Also, since both target and probe were pure-contrast stimuli, their combination by arithmetic addition is also pure-contrast and so low-frequency coding neurons that might respond to a net luminance increase could not discriminate between target and probe in this task. Since the probe and target shared spatial frequencies and since the target was the arithmetic sum of both possible probe orientations (left or right tilted), it was anticipated that any attentional modulation of the neuronal population that responds to targets would be tantamount to modulation of both neural populations that respond to the 2 possible probe orientations. Therefore, it was anticipated that focusing attention on target features without also modulating probe features would be difficult in this task. The target Gabor’s contrast varied among 11 different levels (see Fig. 2*B*), equally spaced between 3% and 40% inclusive (40% was the maximum given the 60% probe). These constituted 11 different difficulty levels, analogous to the range of ring-decrements in the previous experiment. Finally, a different fixation object was employed in this experiment that was designed to maximize gaze stability (Thaler et al. 2013). This was a black circle of diameter 1^o^ with a vertical white cross of equal diameter and arm width of 0.15^o^, and a second black circle in the intersection of the arms of diameter equal to the arm width (see Fig. 2*A*). Note that the reason this fixation object was not used in the previous experiment was to conform to the protocol of the original experiment as closely as possible.

#### Procedure

The goal of experiment 2 was to investigate the impact of performance feedback on C1 modulation and therefore there were 2 conditions: high and low feedback. The procedure for the high-feedback condition was the same as that of the previous experiment except for the following differences. Rather than cue attention on a trial by trial basis, attention was cued at the beginning of each block (and after each of the short breaks within a block). The location cued at the beginning of the block was random but after each short break it was swapped so that each location was cued twice in every block. The reason for this was to give a faster stimulus presentation rate to offset the increase in testing time incurred by the addition of a new task condition. Cueing in this way removed the cue-stimulus interval of the previous experiment and thus the stimulus presentation rate was faster by 750 ms on average. Alpha phase reset was still mitigated through the random inter-stimulus interval of 1533 or 1586 ms (see Fig. 2*D*). Another divergence from the previous experiment was that blocks included 12 null trials in which no stimulus was presented at either location. These were randomly interspersed among stimulus trials and their purpose was to allow us to measure any anticipatory processes that may overlap with stimulus evoked responses in order to subtract these out of the final ERP measurement. However, the number of null trials ultimately proved to be too few and therefore they were not included in any analysis. Excluding null trials, each feedback condition contained the same number of trials as the previous experiment (210 probes per condition).

In the low feedback condition, online feedback was removed. Participants were not told their difficulty level nor were they made aware of them at all. The end of block feedback was limited to their hit, miss and false alarm rates from that block (as in Baumgartner et al. 2018; Alilović et al. 2019), displayed as simple text. In order to keep participants naive to the structure of difficulty level adjustment, all participants underwent the low feedback condition first followed by the high feedback condition, at which point they were informed of the rules governing level adjustment. It was anticipated that participants may notice the changing difficulty levels even in the low feedback condition so they were told before starting that task difficulty may fluctuate throughout the experiment to match their performance but that this was not important to their task and they should ignore it.

### Experiment 3—Target-Features Experiment

#### Participants

Seventeen healthy young adults took part in this experiment (9 females, 14 right handed) with a mean age of 23.5 years (SD = 3.3). They were compensated for their participation either with a lump sum of €20 or with research participation credit if this was required for their course. All participants gave written informed consent, were over the age of 18, had normal or corrected to normal vision and reported no neurological or psychiatric conditions. All operations were approved by the Human Research Ethics for Sciences board of University College Dublin and adhered to the guidelines set out in the Declaration of Helsinki. Two participants who did not exhibit clear C1 signals were removed from C1 analyses (leaving a sample of *N* = 15), but they were retained for other analyses.

#### Stimuli

The goal of this experiment was to investigate whether C1 modulation in a target-detection task is dependent on matching target and probe stimulus features. Therefore, this experiment employed 2 different targets. The matched target was identical to that used in the previous experiment. The unmatched target was a circular luminance disk of radius 0.75^o^ and contrast (with respect to the average luminance of the probe grating) that varied among 11 different difficulty levels equally spaced between 40% and 5%, inclusive (see Fig. 2*C*). It was anticipated that such a target should be encoded primarily by low frequency-tuned neurons and therefore the strategy of selectively targeting low-frequency tuned neurons for attentional modulation was viable (hence, we hypothesized that no modulation of the purely high-frequency probe would take place in this condition). All other stimuli were identical to those of the previous experiment.

For the first 4 participants, the disk stimulus was slightly different. In place of a uniform disk it was a symmetrical 2D Gaussian patch with a sigma value of 0.75^o^, with contrast ranging from 40% to 17.5%. Although subjects reached reasonable performance levels during recordings, they reported difficulty catching on to the task during initial training, hence the transition to a well-defined, more intuitively describable uniform disk target. Since the Gaussian/uniform disk stimuli similarly met the critical design feature of having lower spatial frequency content, we assumed all subjects could be pooled. The hardest contrast level was changed from 17.5% to 5% for the sharp-edge disk in order to maintain overall difficulty and participants reached similar difficulty levels following the change. These first 4 subjects also had a slightly different range of Gabor target contrasts across the 11 difficulty levels, extending from 40% to 3% instead of 40% to 5%, the change being made in order to make the highest difficulty levels more attainable. Importantly, the nontarget probe stimuli to which the VEP responses were measured remained identical.

#### Procedure

The procedure was identical to the high feedback condition of the previous experiment. Since we anticipated that participants would deploy different neural strategies for the 2 types of target, the target-type conditions were carried out in separate sets of consecutive blocks (with condition order counterbalanced across participants) to avoid carry-over of strategy. A practice block was done before starting each target-type condition.

Again, for the first 4 participants, there were some slight procedural deviations. Participants completed 8 blocks of 120 trials per target type (with no null trials). Targets made up 30% of the block and appeared at both locations with equal probability, rather than only at the attended location. The reason for this change was to get a higher proportion of probe trials since target trials were not analyzed. There were also no short breaks during the blocks and the attended location remained the same throughout the block. Finally, the difficulty level was always set to 7 at the beginning of each block (which was changed to reduce the number of trials that participants spent at easier difficulty levels). These discrepancies were considered sufficiently minor to pool across all participants.

#### Data Acquisition (All Experiments)

EEG data were recorded at 512 Hz by an ActiveTwo Biosemi system with 128 scalp electrodes following the Biosemi ABC layout (Biosemi, The Netherlands) and 6 external flat-faced electrodes placed above and below the left eye, on the left and right outer canthi, and on the left and right mastoids. Eye gaze and blinks were monitored via the 4 electrooculograms mentioned above and using an Eyelink Plus 1000 Tower system (SR Research, ON, Canada) recording at 1000 Hz.

#### Data Processing

EEG processing was carried out using a combination of inhouse Matlab scripts (Mathworks) and EEGLAB routines (Delorme and Makeig 2004). Continuous data were low-pass filtered by convolution with a 77-tap hanning-windowed sinc function that provided a 3-dB corner frequency of 35.3 Hz and 83.5 dB of attenuation at 50 Hz (the mains frequency). Subsequently, long segments of persistently high noise in individual channels were identified and interpolated. To identify these, the data were partitioned into short segments of 5 s and for each segment, low-frequency standard deviation (following a 5 Hz low-pass filter) and high-frequency standard deviation (following subtraction of the low-pass filtered data) were calculated and subjected to thresholds of 20 and 10 μV, respectively. Only long segments with a consistent sequence of 7 or more such segments were interpolated. If a short gap of 2 or fewer segments remained between such long segments, they too were interpolated.

Following this, the data were rereferenced to the average of the scalp channels and low-frequency trends were removed. To avoid distortions that can result from typical high-pass filters (Acunzo et al. 2012), we opted instead for a linear detrending approach. For this, we partitioned the data into 4-s segments and detrended each segment by subtracting the line of best fit from every electrode. The partition edges were positioned so as not to coincide with any epoch window, so that the introduced discontinuities would not disrupt epochs. (Therefore, the segments were not always exactly 4 s in duration, but some small deviation from this.) The continuous data were then subjected to in-house artifact detection routines to identify and label time points with electrode “pops,” slow drift, muscle activity and blinks. Pops were identified by the absolute value of a 20 ms-lag amplitude difference exceeding 50 μV. Slow drift and muscle activity artifacts were detected using discrete Fourier transforms of successive one-second partitions. Slow drift was identified if a ratio exceeding 5:1 was found between the maximal frequency-domain amplitudes in the 0–3 and the 3–7 Hz ranges, and muscle activity was identified if a similar ratio in the 3–7 and 20–40 Hz ranges fell below 1:2. Blinks were identified both with the eye-tracking data (for full blinks) and via the difference between the upper and lower vertical electrooculograms (for partial blinks), which was transformed by taking its first derivative (to remove any slow baseline shifts) and applying a 200 ms moving average (to extend blink detection to the periods immediately before and after the main blink where EEG/behavior may still be affected). Data points where this signal exceeded 1 μV were labeled as blinks. Eye tracking data were used to identify saccades, which were defined as gaze displacements over a 10 ms period in excess of 15 times the median across all continuous data. This rather high threshold was chosen due to the strongly skewed nature of these measurements given that these 10 ms intervals frequently contained little to no gaze displacement. The obtained thresholds were 0.66^o^ (SD = 0.16^o^) in experiment 1, 0.55^o^ (SD = 0.24^o^) in experiment 2, and 0.65^o^ (SD = 0.33^o^) in experiment 3. Average trial-loss rates across participants were between 17.2% and 18.8% among all conditions, with a maximum of 52.3% (leaving a minimum of 100 trials per participant per condition). These rates were between 18.8% and 21.4% with a maximum of 52.7% in experiment 2 and between 5.5% and 6.6% with a maximum of 18.6% in experiment 3. Following artifact detection, a copy of the data was subjected to a Laplace transform using the CSD toolbox (Kayser and Tenke 2006), leaving another copy untransformed. (Note that this means that the same data were rejected for artifacts in both raw and CSD-transformed data.)

Finally, the EEG data were segmented into stimulus locked epochs and baseline corrected to a window of −50 to 30 ms. This window was chosen to take into account any potential preparatory activity that may extend beyond initial stimulus presentation. Since the C1 waveform onsets at approximately 50 ms, this baseline window is at a safe distance and should not interfere with the signal. Epochs were rejected if there was a blink during stimulus presentation or if within 100 ms before/after stimulus onset either a saccade took place or median gaze deviation exceeded 1^o^ of visual angle (1.5^o^ in experiment 1 to account for reduced eye tracking precision in the free-sitting position). If a blink did not coincide with stimulus presentation, the epoch was retained but the period containing the blink was excluded from analysis. Similarly, if an EEG pop, slow-wave drift or EMG activity took place within a window of −100 to 150 ms centered on stimulus onset, the epoch was retained but the offending channel was excluded.

### Data Analysis

#### Behavior

Behavioral analyses compared target-type conditions in experiment 3 (Gabor/Disk target), feedback conditions in experiment 2 (high/low feedback), and visual field location for all 3 experiments (upper/lower field). In addition, since the Gabor-target condition in experiment 3 was identical to the high feedback condition of experiment 2, a specific between-groups comparison of these conditions was also conducted (Fig. 6). The measures of interest were response time, difficulty level achieved, accuracy (target hit rates), and *d*′. For response time and difficulty level analyses, each participant’s data were divided into quintile bins on each respective measure and then averaged across subjects, in order to provide richer behavioral data that allow us to examine distributional differences. Accuracy and *d*′ were computed for the trials of each individual difficulty level from level 7 upwards, excluding the lower difficulty levels due to low trial counts and ceiling performance. Within-subjects ANOVA were used for all comparisons, except for those that included between experiments comparisons in which case mixed within- and between-groups ANOVA were used. Greenhouse–Geisser corrected *P*-values were used when the assumption of sphericity was violated.

#### C1 Measurement

In order to account for variability in C1 topography, which may differ as a function of cortical geometry, electrodes were chosen individually for each participant. To do this, epochs corresponding to probes were separated by visual field location (upper/lower) and CSD transformation (on/off) and averaged across all other conditions. Thus, 4 electrode choices were made per participant. Butterfly plots and topographies were generated for each of these waveforms and the electrode that demonstrated the strongest C1 component with as little influence from neighboring components as possible was chosen (see Supplementary Fig. 4 for a visualization of electrode choices across all 3 experiments). C1 topographies were also cross-referenced with the topographies obtained from the PPMVEP procedure to ensure consistency. The reader may notice a greater level of variability in chosen electrode positions for the lower compared with the upper visual field, with lateral choices made more frequently for the lower field. This is likely due to the tendency for lower field stimuli to produce C1 components that are slightly contralateral in topography (Clark et al. 1994; Di Russo et al. 2002; Ales, Yates, et al. 2010; Ales et al. 2013; Kelly, Vanegas, et al. 2013). Once an electrode was chosen, average C1 amplitude was measured between 80 and 90 ms and upper-field amplitudes were multiplied by −1 to account for the flip in polarity between the upper and lower fields. This time window was chosen to align with the peak latency of the C1 waveform in order to maximize the signal-to-noise ratio of C1 amplitude measurements across experiments, and follows recent demonstrations that C1 topographies are uniquely consistent with a V1 source even at peak latency (Kelly, Schroeder, et al. 2013; Kelly, Vanegas, et al. 2013). However, one important objective of this study was to replicate the attentional modulation reported in Kelly et al. (2008), where C1 amplitude was measured between 50 and 80 ms and baseline-corrected to a window between −80 and 0 ms. Therefore, additional analyses for experiment 1 were conducted using these measurement parameters.

C1 amplitudes were assessed for probe trials in each experiment with respect to the factors attention (attended/unattended), stimulus location (upper/lower visual field), experimental condition (high/low feedback in experiment 2; Gabor/Disk target in experiment 3), and CSD transformation (on/off). To allow for meaningful observation of interactions between CSD transformation and other factors given the different units involved (μV for non-CSD and μV/m^2^ for CSD), CSD and non-CSD data were normalized separately by dividing all values by the mean amplitude collapsed across all other factors. Furthermore, a specific comparison was made between the Gabor-target condition of experiment 3 and the high feedback condition of experiment 2, since these conditions employed the same task demands (see Results section). A final analysis aggregated all 3 experiments (collapsing the experimental conditions of experiments 2 and 3) to provide a more highly powered test of the remaining factors (attention, location and CSD). Statistical significance was measured by means of either within-subjects ANOVA or mixed within- and between-subjects ANOVA as appropriate.

#### P1 Measurement

Since there were no behavioral data in the unattended condition, the P1 component was used to gauge the strength of spatial attentional biasing since this component is routinely found to be boosted by spatial attention. Both an earlier peaking contralateral (peaking between 120 and 140 ms) and a later peaking ipsilateral (peaking between 130 and 150 ms) P1 were measured. Since stimulus location differed across subjects, electrodes were chosen for each quadrant of the visual field separately, based on grand average topographies of subjects that had a stimulus location in that quadrant (again, separately for CSD and non-CSD data). P1 amplitudes were then measured in individual subjects for each condition within a window of 120–140 ms for the contralateral P1, and 130–150 ms for the ipsilateral P1. Statistical analysis and data normalization of both P1s were similar to that of the C1.

An exception was made in the case of experiment 1, where the contralateral P1 latency was earlier (peaking at latencies between 90 and 110 ms for lower field stimuli and between 100 and 120 ms for upper-field stimuli), and had already returned to baseline level by the 120–140 ms range. Thus, in this experiment contralateral P1 amplitudes were measured by taking the average in the 90–110 ms range for lower field stimuli and in the 100–120 ms range for upper-field stimuli. Although this change in P1 latency was not expected, variability in P1 latency across experiments is not uncommon and its modulation by spatial attention does not seem to be crucially dependent on its latency (Clark and Hillyard 1996; Hillyard et al. 1998; Di Russo et al. 2003; Fu et al. 2010b; Di Russo et al. 2012). Since P1 latency was not a primary concern in this study, we decided that an investigation of the underlying causes of this latency change was beyond the scope of this paper and therefore we did not seek to clarify this discrepancy. Instead, we adjusted for these cross-experiment differences in latency simply to ensure that we could reliably detect any modulations of the contralateral P1 and thereby gauge the strength of spatial attention deployment. While both the early and late contralateral P1 window were evident in experiment 3, only the late window was evident in experiment 2. For ease of comparison therefore, the later window was chosen for both experiments.

## Results

**Figure 3:**
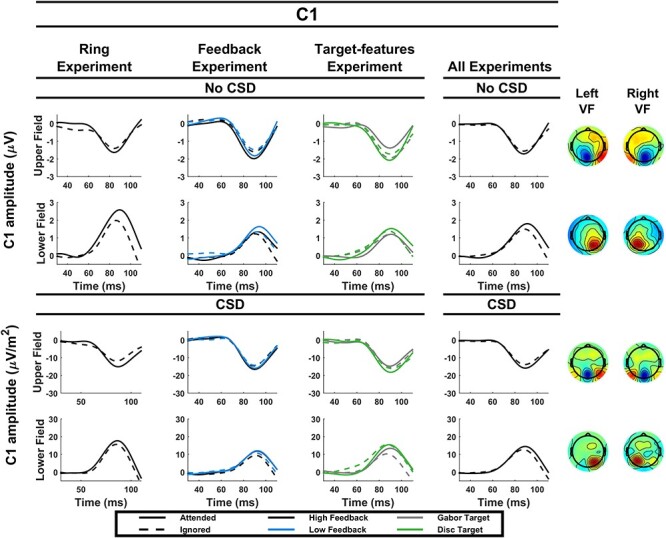
Grand average C1 waveforms. C1 waveforms are shown for each experiment individually (left 3 columns) and for all experiments combined (right column), both with and without CSD transformation and for both the upper and lower visual field. The topographies on the right are based on all experiments combined and show the response to probes for attended and unattended trials collapsed. The left column corresponds to probes shown in the left visual field and the right column corresponds to probes shown in the right visual field. Note, however, that electrodes for C1 measurement were chosen on an individual basis, and therefore these topographies do not precisely reflect the amplitudes of the individually measured C1s used in the waveform plots and statistical tests. For a depiction of the channels used for C1 measurement, the reader is directed to the electrode choices outlined in Supplementary Figure 4.

**Figure 4:**
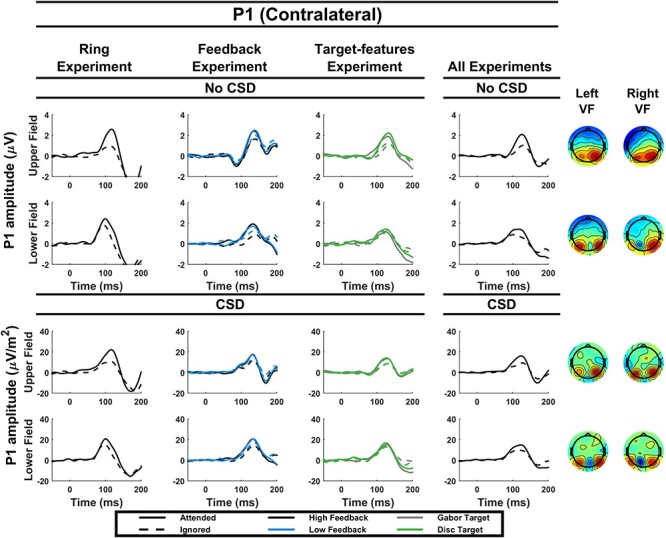
Grand average P1 (contralateral) waveforms. Contralateral P1 waveforms are shown for each experiment individually (left 3 columns) and for all experiments combined (right column), both with and without CSD transformation and for both the upper and lower visual field. The topographies on the right are based on all experiments combined and show the response to probes for attended and unattended trials collapsed. The left column corresponds to probes shown in the left visual field and the right column corresponds to probes shown in the right visual field.

**Figure 5:**
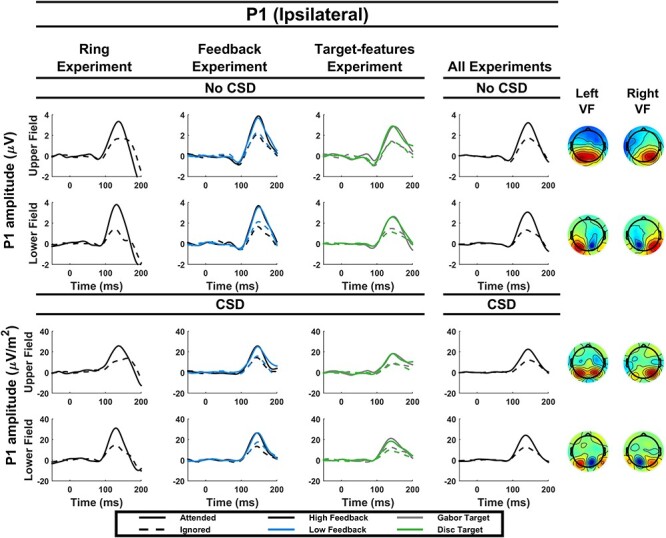
Grand average P1 (ipsilateral) waveforms. Contralateral P1 waveforms are shown for each experiment individually (left 3 columns) and for all experiments combined (right column), both with and without CSD transformation and for both the upper and lower visual field. The topographies on the right are based on all experiments combined and show the response to probes for attended and unattended trials collapsed. The left column corresponds to probes shown in the left visual field and the right column corresponds to probes shown in the right visual field.

We first describe the results of each of the 3 individual experiments, providing all statistical test outcomes in Tables 1–3, and then describe additional analyses conducted across multiple experiments (Tables 4–6). Grand average waveforms in all experiments (including probe trials only as target trials were omitted from analyses) for the upper and lower field, and for CSD- and non-CSD-transformed data are shown for the C1 component (Fig. 3), the contralateral P1 component (Fig. 4), and the ipsilateral P1 component (Fig. 5).

**Table 1:** Results of statistical analyses pertaining to experiment 1

**Experiment 1—Ring Experiment**				
**Behavioural Results**				
**Response Time**	Location	*F(1,15) = 8.63*	*P = 0.0102*	*η_p_^2^ = 0.37*
**VEP Results**				
**C1**	**Attention**	***F(1,14) = 10.64***	***P = 0.0057***	***η***_***p***_^***2***^ ***= 0.43***
	Attention × Location × CSD	*F(1,14) = 6.1*	*P = 0.027*	*η_p_^2^ = 0.30*
***CSD***	**Attention**	***F(1,14) = 11.11***	***P = 0.0049***	***η***_***p***_^***2***^ ***= 0.44***
***no CSD***	Attention	*F(1,14) = 7.99*	*P = 0.0134*	*η_p_^2^ = 0.36*
***lower field***	Attention	*F(1,14) = 4.38*	*P = 0.055*	*η_p_^2^ = 0.24*
	Attention × CSD	*F(1,14) = 3.47*	*P = 0.0836*	*η_p_^2^ = 0.20*
***upper field***	**Attention**	***F(1,14) = 10.62***	***P = 0.0057***	***η***_***p***_^***2***^ ***= 0.43***
**P1 (contralateral)**	**Attention**	***F(1,15) = 24.48***	***P = 0.0002***	***η***_***p***_^***2***^ ***= 0.62***
**P1 (ipsilateral)**	**Attention**	***F(1,15) = 39.94***	***P = 1.4 × 10*** ^***–5***^	***η***_***p***_^***2***^ ***= 0.73***
	Attention × Location × CSD	*F(1,15) = 4.92*	*P = 0.0424*	*η_p_^2^ = 0.25*
***lower field***	**Attention**	***F(1,15) = 20.53***	***P = 0.0004***	***η***_***p***_^***2***^ ***= 0.58***
	Attention × CSD	*F(1,15) = 6.5*	*P = 0.0223*	*η_p_^2^ = 0.30*
***upper field***	**Attention**	***F(1,15) = 12.74***	***P = 0.0028***	***η***_***p***_^***2***^ ***= 0.46***
	Attention × CSD	*F(1,15) = 0.19*	*P = 0.6712*	*η_p_^2^ = 0.01*

Note: Effects are written in bold where *P* < 0.01, in standard text weight when *P* < 0.05, and in small font when *P* < 0.1. For brevity, nonsignificant effects of higher *P*-value are not given, except where we determined that the absence of the effect was important to convey.

**Table 2:** Results of statistical analyses pertaining to experiment 2 (feedback experiment)

**Experiment 2—Feedback Experiment**				
**Behavioural Results**				
**Response Time**	**Feedback**	***F(1,14) = 13.10***	***P = 0.0028***	***η***_***p***_^***2***^ ***= 0.48***
	Feedback × RT-quintile	*F(1.2,16.5) = 5.50*	*P = 0.0273*	*η_p_^2^ = 0.28*
	***Linear Contrast***	***F(1,14) = 15.03***	***P = 0.002***	***η***_***p***_^***2***^ ***= 0.52***
	***Quadratic Contrast***	***F(1,14) = 9.48***	***P = 0.008***	***η***_***p***_^***2***^ ***= 0.40***
	*Cubic Contrast*	*F(1,14) = 5.38*	*P = 0.036*	*η_p_^2^ = 0.28*
**Difficulty Level**	Feedback	*F(1,14) = 8.66*	*P = 0.0107*	*η_p_^2^ = 0.38*
	**Feedback × Difficulty-quintile**	***F(1.4,19.4) = 7.12***	***P = 0.0093***	***η***_***p***_^***2***^ ***= 0.34***
	* Linear Contrast*	*F(1,14) = 8.33*	*P = 0.012*	*η_p_^2^ = 0.37*
**Accuracy**	Feedback	*F(1,14) = 4.89*	*P = 0.0441*	*η_p_^2^ = 0.26*
	Location	*F(1,14) = 5.64*	*P = 0.0324*	*η_p_^2^ = 0.29*
**VEP Results**				
**C1**	Attention	*F(1,14) = 6.78*	*P = 0.0208*	*η_p_^2^ = 0.33*
**P1 (contralateral)**	**Attention**	***F(1,14) = 38.21***	***P = 2.4 × 10*** ^***–5***^	***η***_***p***_^***2***^ ***= 0.73***
	Attention × Feedback	*F(1,14) = 7.90*	*P = 0.0139*	*η_p_^2^ = 0.36*
	Attention × CSD	*F(1,14) = 5.08*	*P = 0.0407*	*η_p_^2^ = 0.27*
	Location × CSD	*F(1,14) = 8.61*	*P = 0.0109*	*η_p_^2^ = 0.38*
**P1 (ipsilateral)**	**Attention**	***F(1,14) = 81.35***	***P = 3.3 × 10*** ^***–7***^	***η***_***p***_^***2***^ ***= 0.85***
	Attention × Feedback	*F(1,14) = 6.17*	*P = 0.0263*	*η_p_^2^ = 0.31*

Note: Where significant interactions are found across quintiles in the behavior, follow-up contrasts are additionally stated to further indicate the nature of the interaction. Effects are written in bold where *P* < 0.01, in standard text weight when *P* < 0.05, and in small font when *P* < 0.1. For brevity, nonsignificant effects of higher *P*-value are not given, except where we determined that the absence of the effect was important to convey.

**Table 3:** Results of statistical analyses pertaining to experiment 3 (target-type experiment)

**Experiment 3—Target-type Experiment**				
**Behavioural Results**				
**Response Time**	**Target-type**	***F(1,16) = 13.67***	***P = 0.002***	***η***_***p***_^***2***^ ***= 0.46***
	**Location**	***F(1,16) = 8.61***	***P = 0.0097***	***η***_***p***_^***2***^ ***= 0.35***
	Target-type × RT-quintile	*F(1.3,21.3) = 4.26*	*P = 0.0415*	***η*** *_p_^2^ = 0.21*
	*Linear Contrast*	*F(1,16) = 6.40*	*P = 0.022*	***η*** *_p_^2^ = 0.29*
	***Location × RT-quintile***	***F(1.4,22.6) = 7.23***	***P = 0.0076***	***η***_***p***_^***2***^ ***= 0.31***
	*Linear Contrast*	*F(1,16) = 8.00*	*P = 0.012*	*η_p_^2^ = 0.33*
	*Quadratic Contrast*	*F(1,16) = 6.40*	*P = 0.022*	*η_p_^2^ = 0.29*
**Accuracy**	Location × Difficulty Level	*F(2.4,38.0) = 3.63*	*P = 0.0294*	*η_p_^2^ = 0.18*
	*Quadratic Contrast*	*F(1,16) = 4.99*	*P = 0.04*	*η_p_^2^ = 0.24*
	*Linear Contrast*	*F(1,16) = 4.79*	*P = 0.044*	*η_p_^2^ = 0.23*
**d’**	Location × Difficulty Level	*F(2.2,35.5) = 3.50*	*P = 0.0366*	*η_p_^2^ = 0.18*
	***Linear Contrast***	***F(1,16) = 10.24***	***P = 0.006***	***η***_***p***_^***2***^ ***= 0.39***
**VEP Results**				
**C1**	Attention	*F(1,14) = 0.04*	*P = 0.8527*	*η_p_^2^ = 0.00*
	**Target-type**	***F(1,14) = 12.49***	***P = 0.0033***	***η***_***p***_^***2***^ ***= 0.47***
	Attention × Target-type × Location	*F(1,14) = 7.07*	*P = 0.0187*	*η_p_^2^ = 0.34*
	Attention × Target-type × CSD	*F(1,14) = 4.69*	*P = 0.0482*	*η_p_^2^ = 0.25*
***Gabor***	Attention × Location	*F(1,14) = 5.67*	*P = 0.032*	*η_p_^2^ = 0.29*
	Attention × CSD	*F(1,14) = 7.26*	*P = 0.0175*	*η_p_^2^ = 0.34*
***Disc***	Attention	*F(1,14) = 1.78*	*P = 0.203*	*η_p_^2^ = 0.11*
***Gabor (no CSD)***	Attention	*F(1,14) = 5.35*	*P = 0.0364*	*η_p_^2^ = 0.28*
	**Attention × Location**	***F(1,14) = 10.75***	***P = 0.0055***	***η***_***p***_^***2***^ ***= 0.43***
***Gabor (CSD)***	Attention	*F(1,14) = 0.77*	*P = 0.3954*	*η_p_^2^ = 0.05*
***Gabor (upper field)***	**Attention × CSD**	***F(1,14) = 8.91***	***P = 0.0098***	***η***_***p***_^***2***^ ***= 0.39***
**P1 (contralateral)**	**Attention**	***F(1,16) = 16.54***	***P = 0.0009***	***η***_***p***_^***2***^ ***= 0.51***
	Attention × Location	*F(1,16) = 4.63*	*P = 0.047*	*η_p_^2^ = 0.22*
	Attention × Location × CSD	*F(1,16) = 8.30*	*P = 0.0109*	*η_p_^2^ = 0.34*
	**Location × CSD**	***F(1,16) = 11.89***	***P = 0.0033***	***η***_***p***_^***2***^ ***= 0.43***
***lower field***	Attention	*F(1,16) = 0.47*	*P = 0.5039*	*η_p_^2^ = 0.03*
	CSD	*F(1,16) = 5.12*	*P = 0.0379*	*η_p_^2^ = 0.24*
***upper field***	**Attention**	***F(1,16) = 23.38***	***P = 0.0002***	***η***_***p***_^***2***^ ***= 0.59***
	CSD	*F(1,16) = 7.87*	*P = 0.0127*	*η_p_^2^ = 0.33*
	Attention × CSD	*F(1,16) = 8.44*	*P = 0.0103*	*η_p_^2^ = 0.35*
**P1 (ipsilateral)**	**Attention**	***F(1,16) = 120.98***	***P = 7.2 × 10*** ^***−9***^	***η***_***p***_^***2***^ ***= 0.88***

Note: Where significant interactions are found across quintiles in the behavior, follow-up contrasts are additionally stated to further indicate the nature of the interaction. Effects are written in bold where *P* < 0.01, in standard text weight when *P* < 0.05, and in small font when *P* < 0.1. For brevity, nonsignificant effects of higher *P*-value are not given, except where we determined that the absence of the effect was important to convey.

**Table 4:** Results of statistical analyses comparing the high-feedback condition of experiment 2 with the Gabor-target condition of experiment 3

**Comparing Experiments 2 and 3**				
**Behavioral Results**				
**Response Time**	Location	*F(1,30) = 4.99*	*P = 0.033*	*η_p_^2^ = 0.14*
	Experiment	*F(1,30) = 3.76*	*P = 0.062*	*η_p_^2^ = 0.11*
**Difficulty Level**	**Difficulty-quintile × Experiment**	***F(4,120) = 5.84***	***P = 0.008***	***η***_***p***_^***2***^ ***= 0.16***
	*Linear Contrast*	*F(1,30) = 6.01*	*P = 0.02*	*η_p_^2^ = 0.17*
	***Quadratic Contrast***	***F(1,30) = 8.55***	***P = 0.007***	***η***_***p***_^***2***^ ***= 0.22***
**Accuracy**	Difficulty Level × Experiment	*F(10,300) = 3.78*	*P = 0.016*	*η_p_^2^ = 0.11*
	***Linear Contrast***	***F(1,30) = 8.46***	***P = 0.007***	***η***_***p***_^***2***^ ***= 0.22***
	**Experiment**	***F(1,30) = 8.86***	***P = 0.006***	***η***_***p***_^***2***^ ***= 0.23***
**d’**	Experiment	*F(1,30) = 6.31*	*P = 0.018*	*η_p_^2^ = 0.17*
**VEP Results**				
**C1**	Attention	*F(1,28) = 2.11*	*P = 0.157*	*η_p_^2^ = 0.07*
	Attention × Experiment	*F(1,28) = 5.74*	*P = 0.023*	*η_p_^2^ = 0.17*
	Attention × Experiment × CSD	*F(1,28) = 5.62*	*P = 0.025*	*η_p_^2^ = 0.17*
	Attention × Experiment × Location	*F(1,28) = 4.36*	*P = 0.046*	*η_p_^2^ = 0.14*
	Attention × Experiment × Location × CSD	*F(1,28) = 3.64*	*P = 0.067*	*η_p_^2^ = 0.12*
***no CSD***	**Attention × Experiment**	***F(1,28) = 9.71***	***P = 0.004***	***η***_***p***_^***2***^ ***= 0.26***
	**Attention × Experiment × Location**	***F(1,28) = 7.77***	***P = 0.009***	***η***_***p***_^***2***^ ***= 0.22***
***CSD***	Attention	*F(1,28) = 6.13*	*P = 0.02*	*η_p_^2^ = 0.18*
	Attention × Experiment	*F(1,28) = 0.44*	*P = 0.515*	*η_p_^2^ = 0.01*
**P1 (contralateral)**	Attention × Experiment	*F(1,30) = 7.52*	*P = 0.01*	*η_p_^2^ = 0.20*
	Attention × Experiment × Location × CSD	*F(1,30) = 4.88*	*P = 0.035*	*η_p_^2^ = 0.14*

Note: Where significant interactions are found across quintiles in the behavior, follow-up contrasts are additionally stated to further indicate the nature of the interaction. Effects are written in bold where *P* < 0.01, in standard text weight when *P* < 0.05, and in small font when *P* < 0.1. For brevity, nonsignificant effects of higher *P*-value are not given, except where we determined that the absence of the effect was important to convey.

**Table 5:** Results of statistical tests collapsed across all 3 experiments

**All Experiments Collapsed**				
**C1**	**Attention**	***F(1,45) = 12.96***	***P = 0.001***	***η***_***p***_^***2***^ ***= 0.24***
	Attention × Experiment	*F(2,45) = 2.98*	*P = 0.062*	*η_p_^2^ = 0.12*
	Attention × Experiment × CSD	*F(2,45) = 3.03*	*P = 0.059*	*η_p_^2^ = 0.13*
	Attention × Experiment × CSD × Location	*F(2,45) = 3.13*	*P = 0.054*	*η_p_^2^ = 0.13*
**P1 (contralateral)**	**Attention**	***F(1,45) = 69.01***	***P = 1.2 × 10*** ^***−10***^	***η***_***p***_^***2***^ ***= 0.60***
	**Attention × CSD**	***F(1,45) = 8.35***	***P = 0.006***	***η***_***p***_^***2***^ ***= 0.16***
	Attention × CSD × Experiment	*F(1,45) = 4.71*	*P = 0.014*	*η_p_^2^ = 0.17*
	Attention × Location	*F(1,45) = 6.15*	*P = 0.017*	*η_p_^2^ = 0.12*
	Attention × Location × CSD	*F(1,45) = 6.57*	*P = 0.014*	*η_p_^2^ = 0.13*
	Attention × Location × CSD × Experiment	*F(2,45) = 3.24*	*P = 0.047*	*η_p_^2^ = 0.13*
***no CSD***	**Attention**	***F(1,45) = 69.62***	***P = 1.1 × 10*** ^***−10***^	***η***_***p***_^***2***^ ***= 0.61***
	**Attention × Location**	***F(1,45) = 8.04***	***P = 0.007***	***η***_***p***_^***2***^ ***= 0.15***
***CSD***	**Attention**	***F(1,45) = 50.95***	***P = 6.4 × 10*** ^***−9***^	***η***_***p***_^***2***^ ***= 0.53***
	Attention × Location	*F(1,45) = 2.91*	*P = 0.095*	*η_p_^2^ = 0.06*
***lower field***	**Attention**	***F(1,45) = 15.26***	***P = 0.0003***	***η***_***p***_^***2***^ ***= 0.25***
**P1 (ipsilateral)**	**Attention**	***F(1,45) = 194.47***	***P = 6.0 × 10*** ^***−18***^	***η***_***p***_^***2***^ ***= 0.81***
	Attention × CSD	*F(1,45) = 4.45*	*P = 0.04*	*η_p_^2^ = 0.09*
	Attention × CSD × Location × Experiment	*F(2,45) = 3.94*	*P = 0.027*	*η_p_^2^ = 0.15*
***no CSD***	**Attention**	***F(1,45) = 159.64***	***P = 2.1 × 10*** ^***−16***^	***η***_***p***_^***2***^ ***= 0.78***
***CSD***	**Attention**	***F(1,45) = 202.92***	***P = 2.8 × 10*** ^***−18***^	***η***_***p***_^***2***^ ***= 0.82***

Note: Effects are written in bold where *P* < 0.01, in standard text weight when *P* < 0.05, and in small font when *P* < 0.1. For brevity, nonsignificant effects of higher *P*-value are not given, except where we determined that the absence of the effect was important to convey.

### Experiment 1—Ring Experiment (Reproduction of Original)

#### Behavior

Participants were challenged during this task as indexed by the fact that the top 20% of participants’ trials fell below ceiling (which was level 11) at an average difficulty level of 9.4 (SD = 0.6). Responses were faster in the lower visual field (*P* = 0.01) but there was no effect of visual field on accuracy.

#### C1 Component

One participant who did not show a clear C1 waveform in the lower visual field without CSD transformation was excluded from analysis, leaving a total of *N* = 15 (they were, however, included for the later P1 analyses). A main effect of attention (*P* = 0.006) indicated that attention boosted the C1 component. In order to take into account the unequal overlap with the P1 component for the upper and lower visual fields, we repeated this analysis for each visual field separately (see Table 1). This indicated that the effect was significant in the upper visual field but marginal in the lower visual field (*P* = 0.055) It is unlikely that this effect was driven by deviations in eye gaze as, in addition to rejecting trials with gaze deviation beyond 1.5^o^, we measured average gaze deviation in the direction of the attended location across trials for each participant and the maximum such average was 0.26^o^, which is small compared with the eccentricity of the stimulus (4^o^).

In order to directly test for a replication of the attentional modulation reported by Kelly et al. (2008), we repeated this analysis using non-CSD transformed data only and using a C1 measurement window of 50–80 ms and a baseline window of −80 to 0 ms, as was done in the original study. In this window, the effect of attention was nonsignificant (*F*(1,14) = 0.85, *P* = 0.37) here. However, directly comparing the data of the original study with this new replication dataset in a single mixed ANOVA yielded no significant interaction between experiment and attention using either the 50–80 ms window (*F*(1,24) = 3.28, *P* = 0.08) or the 80–90 ms window (*F*(1,24) = 2.35, *P* = 0.14), while the main effect of attention remained significant (*F*(1,24) = 10.19, *P* = 0.004). A comparison of the present waveforms with those of the original study is provided in Supplementary Figure 5.

Since there was marginal evidence that might suggest a weaker effect in the new data in the earlier timeframe, we performed a formal statistical comparison of the onset latency of the attention modulation in the 2 datasets. To do this, we collapsed upper- and lower-field waveforms (multiplying the upper waveforms by −1 to account for polarity inversion), and taking jackknife samples (leaving a single subject out in turn), found the latency at which the mean attentional modulation reached 30% of its eventual maximum level. This modulation onset was estimated at 60 ms in the original dataset and at 66 ms in the present dataset, a difference that was not statistically significant (*t*(15.59) = 0.6, *P* = 0.56). Collapsing experiments, the onset latency of modulation was between 58.7 and 72.3 ms with 95% confidence.

#### P1 Components

Attention boosted both the contralateral P1 (*P* = 0.0002) and the ipsilateral (*P* = 1.4 × 10^−5^) P1 components.

### Experiment 2—Feedback Experiment

#### Behavior

Two subjects were excluded from this analysis as they did not complete the second half of the experiment (the high feedback condition), leaving a total of *N* = 15. These participants were also excluded from the later VEP analyses.

The task in this experiment was challenging too, with the top 20% of trials spent at an average level of 9.5 (SD = 0.8). Performance improved in the high feedback condition overall: Responses became faster (*P* = 0.003), higher difficulty levels were achieved (*P* = 0.01), and accuracy was improved (*P* = 0.04).

Because the low feedback condition was always carried out first, it is possible that effects of feedback may be driven by effects of learning. In an effort to disentangle the 2 possibilities, we divided the experiment into 4 quarters with the first 2 stemming from the low feedback condition (see Supplementary Fig. 6). We then compared the jump in performance between the second and third quarters (which differ in terms of both feedback and time on task) with the jumps between the first 2 and the last 2 quarters (which differ in terms of time on task only). The rationale was that an effect of feedback itself should manifest as a large jump at the transition between low and high feedback with relatively smaller jumps between the other quarters. Indeed, the jump at transition for response time was significantly larger than the other 2 jumps (*F*(1,14) = 11.72, *P* = 0.004, μ*_p_*^2^  = 0.46), reflecting an effect of feedback. However, jumps in difficulty level between quarters did not differ significantly in their magnitude (*F*(1,14) = 1.05, *P* = 0.32, μ*_p_*^2^ = 0.07).

#### C1 Component

A main effect of attention indicated that attention boosted the C1 (*P* = 0.02). However, this effect did not interact with feedback condition (*P* > 0.1), or indeed any other factor. Five participants in this experiment had average gaze deviations toward the attended location in excess of 0.3^o^, of which one had already been excluded on the basis of not completing the high feedback condition. To rule out a potential role of eye gaze deviation in generating this result, we repeated the analysis with these additional 4 participants excluded, which retained the significant effect of attention on the C1 (*F*(1,10) = 7.22, *P* = 0.02). Moreover, there was no correlation between the magnitude of gaze deviation toward the cued location and attentional modulation of the C1, with the relationship in fact trending in the opposite direction (*r* = −0.28, *P* = 0.31).

Again, in order to take into account the unequal overlap with the P1 component for the upper and lower visual fields, we repeated this analysis for each visual field separately. Since exclusion of the 4 participants whose average gaze deviation exceeded 0.3^o^ did not affect the above results, they were included in this analysis. In the upper visual field, the effect of attention was exhibited (*F*(1,14) = 5.7, *P* = 0.03). However, in the lower visual field, the effect was marginal (*F*(1,14) = 4.0, *P* = 0.06).

#### P1 Components

Both the contralateral (*P* = 2.4 × 10^−5^) and ipsilateral (*P* = 3.3 × 10^−7^) P1 components were boosted by attention. Furthermore, feedback level interacted with this effect, demonstrating that attention boosted both the contralateral P1 (*P* = 0.01) and the ipsilateral P1 (*P* = 0.03) more in the high feedback condition than in the low feedback condition.

### Experiment 3—Target-Type Experiment

#### Behavior

In this experiment, participants achieved an average level in their top 20% of trials of 9.8 (SD = 0.6). As in experiment 1, responses were faster in the lower visual field (*P* = 0.01). Behavior also differed by target type. Responses were slower when detecting a Gabor target than when detecting a disk target (*P* = 0.002).

#### C1 Component

Two participants who did not show a clear C1 component in either or both visual fields were excluded from this analysis, leaving a total of 15. They were, however, retained for the subsequent P1 analyses.

Attention did not significantly modulate the C1 component in this experiment overall (*P* = 0.85). However, there were significant interactions indicating that the impact of attention depended on target-type, visual field location, and CSD (see Table 3 and Fig. 3). Follow-up analyses revealed that while attention did not modulate the C1 for either target type in either visual field when CSD transformation was applied, attention significantly suppressed the C1 in the upper visual field when the target was a Gabor (*P* = 0.005), but only when no CSD transformation was applied. Furthermore, a significant main effect of target-type indicated that C1s were smaller when the target was a Gabor than when the target was a disk (*P* = 0.003).

It is unlikely that this effect was driven by deviations in eye gaze as, in addition to rejecting trials with gaze deviation beyond 1^o^, we measured average gaze deviation in the direction of the attended location across trials for each participant and the maximum such average was 0.11^o^, which is small compared with the eccentricity of the stimulus (6^o^).

To ensure that this surprising effect was not driven by the 4 participants who underwent a slightly different protocol (see Methods—Experiment 3), we plotted C1 amplitudes across all conditions for all individual participants in order to determine whether any outliers were present among those 4 participants (the small sample size of *N* = 4 precluded any formal between-groups comparison). No outliers were apparent, as shown in Supplementary Figure 9 (see Supplementary Figures 7 and 8 for similar plots pertaining to experiments 1 and 2). Indeed, the same numerical trend of attentional suppression of the upper-field C1 was present in these 4 participants, as it was in the majority of all participants.

#### P1 Component (Contralateral)

There was a main effect of attention on the contralateral P1 (*P* = 0.0009), but this was accompanied by an interaction with location (*P* = 0.047) and follow up analyses revealed that while the effect was significant in the upper field (*P* = 0.0002), it was nonsignificant in the lower field (*P* = 0.5). There was also a significant location by CSD interaction, reflecting a reduction of the upper-field P1 amplitude by CSD transformation (*P* = 0.003). A three-way interaction between attention, location and CSD further indicated that CSD reduced the size of the attention effect in the upper field. Note that these results mirror the C1 suppression that was eliminated by CSD transformation, which we return to in the discussion.

**Table 6:** Results of statistical tests comparing different latency windows for measurement of C1 amplitudes

**C1 by Latency Range**				
**C1 latency**				
**80 – 90 ms**	**Attention**	***F(1,45) = 12.96***	***P = 0.001***	***η***_***p***_^***2***^ ***= 0.24***
	Attention × Experiment	*F(2,45) = 2.98*	*P = 0.062*	*η_p_^2^ = 0.12*
	Attention × Experiment × CSD	*F(2,45) = 3.03*	*P = 0.059*	*η_p_^2^ = 0.13*
	Attention × Experiment × CSD × Location	*F(2,45) = 3.13*	*P = 0.054*	*η_p_^2^ = 0.13*
**75 – 85 ms**	Attention	*F(1,45) = 6.41*	*P = 0.015*	*η_p_^2^ = 0.13*
	Attention × Experiment	*F(2,45) = 2.95*	*P = 0.063*	*η_p_^2^ = 0.12*
	Attention × Experiment × CSD	*F(2,45) = 2.76*	*P = 0.75*	*η_p_^2^ = 0.12*
**70 – 80 ms**	Attention	*F(1,45) = 1.39*	*P = 0.244*	*η_p_^2^ = 0.03*

Note: Effects are written in bold where *P* < 0.01, in standard text weight when *P* < 0.05, and in small font when *P* < 0.1. For brevity, nonsignificant effects of higher *P*-value are not given, except where we determined that the absence of the effect was important to convey.

#### An Early Onset of the Contralateral P1

Upon visual inspection of the contralateral P1 waveforms for experiment 3, it appeared that the P1 attention effect had a particularly early onset in the specific case of the Gabor target in the upper field, without CSD transformation (Fig. 4, top of third column)—the same condition that showed a paradoxical reverse C1 modulation. To explore the possibility that the latter effect may be driven by the overlapping P1 effect, we ran an ANOVA on amplitudes measured from P1 electrodes but in the early 80–90 ms time range coinciding with the C1. This revealed an attention × target-type interaction (*F*(1,16) = 5.11, *P* = 0.04, μ*_p_*^2^ = 0.24), which was followed by a Gabor target-specific interaction among attention, location and CSD (*F*(1,16) = 5.8, *P* = 0.03, μ*_p_*^2^ = 0.27). This indicates that the P1 onset early in the upper visual field specifically for the Gabor target without CSD transformation.

#### P1 Component (Ipsilateral)

The ipsilateral P1 was boosted by attention (*P* = 7.2 × 10^−9^) and there were no accompanying interactions.

### Comparing Experiments 2 and 3

The high feedback condition of experiment 2 and the Gabor-target condition of experiment 3 were in fact identical protocols, except that participants in experiment 2 had all previously completed the low feedback condition, whereas participants in experiment 3 had high feedback from the start. It was therefore surprising that these 2 conditions displayed different patterns of VEP effects. To establish whether this reflects a statistically reliable difference between the experiments, we conducted additional ANOVAs on the data of just those matching conditions with the between-groups factor of experiment included.

#### Behavior

**Figure 6:**
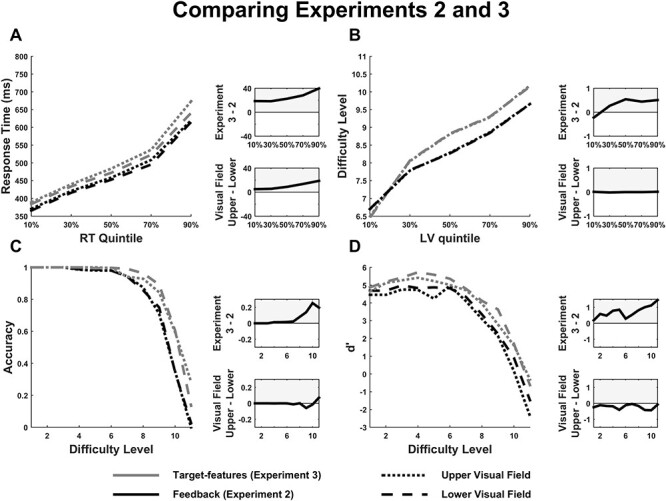
Behavior compared between the Gabor-target condition of experiment 3 and the high feedback condition of experiment 2. Response times (*A*), difficulty level (LV) achieved (*B*), target accuracy/hit rate (*C*), and d′ (*D*) are depicted, broken down by experiment and visual field location. Adjacent to each figure are difference plots showing the difference between the experiments (top) and between visual field locations (bottom). Behavioral performance in each individual experiment is shown in Supplementary Figure 10.

Overall, Gabor-target detection performance in experiment 3 was better than in experiment 2 (see Fig. 6). Note that this is despite the fact that the first 4 participants of experiment 3 underwent a slightly more difficult version of the experiment (see Methods). Participants spent more time at the highest difficulty levels (Quadratic contrast, *P* = 0.024), their target accuracy was superior especially at these higher levels (linear contrast *P* = 0.007), and target sensitivity (*d*′) was also higher (*P* = 0.02). Response time patterns appeared not to reliably differ across experiments, in that no main effect or interactions involving experiment reached significance.

#### VEP Components

As expected, there was a significant experiment × attention interaction (*P* = 0.02) due to the C1 attention effect being present in the feedback experiment but not in the target-type experiment. This interaction was modified by further interactions with CSD and location (see Table 4), again reflecting the specificity of the reverse-modulation effect to the upper field, Gabor-target condition without CSD in experiment 3.

For the contralateral P1, there was a significant attention by experiment interaction (*P* = 0.01) indicating a larger effect of attention on the P1 in the feedback experiment. There was also a significant four-way interaction across all conditions, indicating 2 things: (1) CSD reduced the attention effect for the upper field in the target-features experiment more so than in the feedback experiment, mirroring the similar removal of C1 suppression, and (2) attention boosted the P1 across the board except for the lower visual field in the target-type experiment.

### Collapsing Across all Experiments

Although each experiment had a distinct goal, all 3 employed highly similar target detection tasks of comparable difficulty, presenting an opportunity for a highly powered statistical test by pooling data across experiments. To do this, the high and low feedback conditions of experiment 2 were averaged and the Gabor and disk target conditions of experiment 3 were averaged, leaving attention, visual field location, and CSD transformation as within-subjects factors, and experiment as a between-groups factor. This yielded a sample size of *N* = 45 for the C1 analysis and *N* = 48 for the P1 analyses.

#### C1 Component

A main effect of attention indicated that spatial attention boosted the C1 overall (*P* = 0.001). This was accompanied by no significant interaction, though a number of interactions approached significance (see Table 5). In order to take into account the unequal overlap with the P1 component for the upper and lower visual fields, we repeated this analysis for each visual field separately. In this vein, the above main effect of attention was present both in the lower (*F*(1,42) = 6.36, *P* = 0.02) and in the upper visual field (*F*(1,42) = 9.52, *P* = 0.004).

#### P1 Component (Contralateral)

Attention boosted the contralateral P1 overall (*P* = 1.2 × 10^−10^), and this effect interacted with experiment, CSD transformation and visual field (see Table 5). Follow up analyses indicated that the attention effect was diminished by CSD and was smaller in the lower visual field.

It is commonly stated that the overlap between the lower field C1 component and the contralateral P1 component can make interpretation of lower field C1 effects difficult (Di Russo et al. 2003; Fu et al. 2010a; Ding et al. 2014; Dassanayake et al. 2016). Therefore, we assessed the extent of this issue in the present dataset by testing for amplitude effects at the P1 electrodes but during the same time window as that used to measure the C1 (80–90 ms). In this time window, a small but significant effect of attention on the P1 remained (*F*(1,45) = 4.8, *P* = 0.03) but an interaction between attention and component indicated that the attention effect was stronger at the C1 than the P1 electrodes in this time range (*F*(1,42) = 5.56, *P* = 0.02).

#### P1 Component (Ipsilateral)

Attention boosted the ipsilateral P1 overall (*P* = 6.0 × 10^−18^).

### C1 by Latency Range

One of the central questions relating to C1 modulation by attention pertains to the time-frame in which the modulation occurs. To address this, we progressively moved the C1 measurement window back by 5 ms at a time until the effect was no longer significant when collapsed across experiments (see Table 6). Measured between 75 and 85 ms, the C1 remained stronger at attended locations (*P* < 0.05). However, the effect was lost between 70 and 80 ms (*P* > 0.1).

Finally, although C1 amplitudes are typically measured using a common latency window across all participants, this ignores individual variability in the latency of the C1 peak, which may vary due to variability in the latency of the underlying neural activity, but also simply as a result of differences in the relative strength or latency of overlapping components (e.g., Foxe and Simpson 2002). Therefore, we individually chose C1 latency ranges by visual inspection and compared these C1 amplitudes with those measured using the common latency (see Supplementary Fig. 11 and Supplementary Table 1). C1s were larger across the board with the individual latency ranges (*P* = 8.6 × 10^−5^), especially in the lower visual field (*P* = 0.02). Most importantly, we confirmed that the effects of attention reported based on the common latency window are the same qualitatively using individual latency windows.

## Discussion

Despite a robust past demonstration of C1 modulation by spatial attention (Kelly et al. 2008), 2 recent, well-implemented replication attempts (Baumgartner et al. 2018; Alilović et al. 2019) failed to reproduce the result. This hints at the possibility that subtle differences between the experiments may shine light on fundamental principles of the operation of spatial attention during early visual processing. Here, we sought to more closely replicate the original experiment, and hence potentially the effect, reported by Kelly et al. (2008) in experiment 1. In experiment 2, we tested the hypothesis that the strength (or even presence at all) of a C1 spatial attention modulation is influenced by the extent of performance feedback that is given to participants. Finally, in experiment 3, we tested the hypothesis that a C1 modulation occurs in a spatially cued target detection task when the target and probe share stimulus features but that the effect is eliminated if the target is identifiable by stimulus features that are absent in the probe. This paper presents the first systematic examination of the impact that target stimulus-features have on the emergence of C1 modulations. Others have demonstrated that the C1 attention effect is moderated by perceptual load (e.g., Fu et al. 2010a, 2010b) and there have been mixed reports regarding the impact of attentional load (Rauss et al. 2012; Ding et al. 2014) but to date there has been no VEP investigation into the impact of the specific task demands imposed by the conjunction of stimulus features held by the target stimulus and by the probe stimulus used to measure the attentional effect.

Collapsing across all 3 experiments, yielding a highly powered test (*N* = 45), we found that, overall, spatial attention boosted the C1, by ~11%. This was accompanied by the typically observed modulation of the P1 component (52% and 94% for the contralateral and ipsilateral components, respectively), thus validating that attention was indeed strongly deployed. Crucially, the C1 effect was also present individually in experiment 1, thereby replicating the original study (Kelly et al. 2008), although a later primary measurement window was used here. While the effect was nonsignificant when using the original 50–80 ms window, directly comparing the original and new datasets in this window, we found no significant difference in either the magnitude or the onset latency of the attention modulation. Although this was necessarily a between-groups comparison and was thus underpowered to detect more subtle differences, the modulation onset latency was numerically similar (60 compared with 66 ms), and the 95% confidence interval for the modulation onset of the pooled data (*N* = 26) spanned 58–72 ms, a range that is well within the rising phase of the initial afferent response reflected in C1 (Foxe and Simpson 2002). Finally, it is unlikely that this effect was driven by overlap with the P1 component as the effect was also present when considering the upper visual field alone (where the modulation runs counter to that of the P1).

Attention boosted the C1 in experiment 2 as well. Again, this was also true when considering the upper visual field in isolation, arguing against the possibility that this was due to overlap with the P1 component. This effect represents a second reproduction of the C1’s modulation by spatial attention in a task condition that differed in several ways, including the different target stimuli employed, the different size and eccentricity of the stimuli, the different relative levels of background luminance, and different attentional cueing regimes (blocked rather than trial by trial). This suggests that such modulation is not confined to some specific, arbitrary set of task conditions. Although attention boosted the C1 in this experiment, the extent of performance feedback provided did not have any effect on the size of this modulation. This was despite the fact that high feedback increased the attention effect of both the contralateral and ipsilateral P1 components. Because the high feedback condition was always carried out second, it is imperative to consider the possibility that an effect of feedback may in fact be an effect of learning. Indeed, an analysis of behavioral improvement throughout experiment 2 suggested that both learning and feedback had an influence. While response time improvements were driven mostly by feedback, the improvements in difficulty level were less clearly distinguishable from one arising from learning alone. It is difficult to disentangle the influence of feedback from learning since a primary effect of feedback is to enhance learning, and we do not know what the learning curve would have looked like in the absence of the change of feedback. Thus, we cannot say for certain whether the effect of feedback condition on the P1 attention effect was driven by an improvement due merely to time and/or to the better feedback. It is interesting to note that the attention effect presented as a reduction in unattended P1 amplitude under high feedback without any change in attended P1 size, which mirrors previous research in the perceptual learning domain that demonstrates improved suppression of the P1 to distractor stimuli (Berry et al. 2009) but no change in P1 amplitude to target stimuli (Pourtois et al. 2008).

By contrast to the first 2 experiments, attention did not boost the C1 in experiment 3. In fact, it was actually suppressed in the upper visual field, but with no modulation in the lower visual field and no modulation at all for the condition in which the target was a disk. While this absence of a modulation for the disk-target condition was consistent with our hypothesis that modulation would not be observed when target features were not matched to the probe, the suppressive effect in the condition with overlaid grating targets was unexpected. Although our available data do not offer direct means to definitively resolve this, below we discuss several results that together point to one potential explanation, namely that the subjects of experiment 3 learned a superior strategy for performing this texture-based task involving early enhancement of area V2, whose polarity on the scalp opposes that of V1.

The upper-field C1 suppression was accompanied by the conspicuous absence of a contralateral P1 modulation in the lower visual field, and both of these findings were unique to experiment 3, despite the fact that the matched-target condition in this experiment was ostensibly identical to the high feedback condition of experiment 2. Although identical in physical stimulation, the participants encountered these conditions in different contexts. In experiment 2, they performed the condition straight after a set of blocks with no feedback, whereas in experiment 3, participants had feedback from the start of the experiment and these latter participants performed significantly better. There is evidence from the perceptual learning literature that while feedback-free learning does take place, it can be inferior to feedback-assisted learning in some contexts and this shortfall can sometimes fail to be corrected by the subsequent introduction of feedback (Mckee and Westhe 1978; Shiu and Pashler 1992; Crist et al. 1997; Herzog and Fahle 1997; Petrov et al. 2006). This suggests that the superior performance observed in experiment 3 is likely to be a result of improved learning that was facilitated by the richer feedback provided from the start. In particular, these subjects may have learned a superior strategy tailored to the properties of these stimuli and their encoding in the visual system. Although we initially assumed that discrimination decisions in this task would be derived from low-level neurons tuned to either of the individual orientations, the superimposition of the 2 orientations within a target creates a texture pattern from its consistent sequence of local luminance variations (e.g., Beck 1983; Rosenholtz 1999). V2 has been shown to be more selective for textural information than V1 (Merigan et al. 1993; Freeman et al. 2013; Yu et al. 2015; Ziemba et al. 2016), and thus may have been selectively utilized to distinguish targets from single-orientation probe stimuli. V2 also opposes V1 geometrically for much of the visual field (and principally at the same spatial locations as were typically chosen in this study), and would therefore produce scalp potentials of opposite polarity to those originating in V1 (Schroeder et al. 1991; Schroeder et al. 1998; Wandell et al. 2009; Ales et al. 2013). Furthermore, while V1 is generally thought to be the primary source of the C1 component, it is unlikely that it is the sole contributor and there are suggestions that V2 (and possibly V3) could make a comparably strong contribution (e.g., Ales, Yates, et al. 2010). This raises the possibility that the observed suppression of the C1 by attention in this task could be explained by a spatial modulation of V2 rather than V1. There are a number of aspects of the results presented here that are consistent with such a view: (1) CSD transformation eliminated the C1 suppression effect but it did not eliminate any of the other attentional modulations. This suggests encroachment from an overlapping signal (itself modulated by attention), which was mitigated by the improved spatial resolution of CSD. Given that V2 and V1 produce potentials of reverse polarity and both are purported to contribute to the C1 (though to different extents), V2 is a good candidate source for such an overlapping signal. (2) C1 responses in experiment 3 were significantly larger when the target was a disk than when the target was a Gabor. Importantly, the stimulus eliciting the measured C1 responses was identical in both of these cases, suggesting an overall task-based modulation of the C1. Since the disk target is not amenable to the same texture-detection strategy as is the Gabor target, V2 may have been preferably boosted during the Gabor-detection task, thereby reducing overall C1 amplitude. (3) Attentional modulation of the P1 component in the upper visual field began at an early latency that coincided with the C1 time range and this early onset was eliminated by CSD transformation (which did not eliminate the modulation at its later latency). Again, this implies that the early portion of the modulation stemmed from a separate source to that of the P1. This early, positive-going modulation in the upper visual field is again consistent with a V2/V3 source. What is more, upper field P1s were larger than lower field P1s, but CSD transformation brought them to an equal amplitude. The P1 would tend to be boosted by V2/V3 in the upper field but suppressed in the lower field so this finding is also consistent with V2 recruitment in this task. (4) Both the absence of a lower field counterpart to the C1 suppression effect and the conspicuous absence of a lower field P1 modulation (to mirror the strong, early, upper field modulation) could be explained by the two modulations canceling out since they would have had opposite polarity. Thus, V2 modulation offers a unifying explanation for a wide range of the peculiar VEP effects observed in this experiment.

While the above considerations make V2 overlap a tantalizing explanation for the observed C1 suppression and contralateral P1 nonmodulation, there are a number of caveats that need to be taken into account. While the absence of a lower field C1 suppression effect could be explained by a cancelation of V2 and P1 modulations, it is still surprising that the lower field P1 modulation would not appear at any latency. What’s more, the lower field P1 was not modulated in the disk detection task either, but in this case, there was no accompanying upper-field C1 suppression. Also, while CSD transformation eliminated the C1 suppression effect, it did not introduce a lower field P1 modulation, which is difficult to reconcile with the idea that the 2 effects are linked via overlap with V2. All of this suggests that the upper field C1 suppression and lower field P1 nonmodulation may not actually be linked, with C1 suppression representing a target-specific effect and the separate P1 nonmodulation representing a target nonspecific effect. This leaves us with a convincing suggestion that attention was allocated asymmetrically between the upper- and lower-visual field in this experiment. Indeed, performance in texture discrimination tasks varies considerably across the visual field, with performance maximal at intermediate eccentricities where spatial resolution is ideally suited to aggregate the local luminance fluctuations that constitute texture (Yeshurun and Carrasco 1998; Carrasco 2011). Furthermore, there is a performance discrepancy in these tasks between the upper- and lower-visual field whereby performance peaks at greater eccentricities in the lower visual field, due to its generally higher spatial resolution at all eccentricities (Talgar and Carrasco 2002; Carrasco 2011). In fact, inspecting Figure 2*C* in (Talgar and Carrasco 2002), it seems that the eccentricity of our stimuli (6^o^) falls close to both cued and uncued peak performance for the lower visual field, but that for the upper visual field, uncued performance peaks at lower eccentricities (between 3^o^ and 4^o^) while cued performance peaks between 4^o^ and 5^o^. This suggests that the upper visual field may have elicited a greater need for attentional modulation than the lower visual field in our task. While it may be difficult to draw a numerical parallel across these experiments in terms of the performance functions of eccentricity given that Talgar and Carrasco used different stimuli to ours and placed them along the vertical meridian, the idea is nevertheless supported by the fact that performance in our task was inferior in the upper visual field (suggesting a greater need for attentional improvement). However, despite considerable performance variability across the visual field, sustained attention appears to improve performance comparably at all eccentricities (Yeshurun et al. 2008) and to our knowledge there is no evidence of spatial attentional asymmetries between the upper and lower visual field in texture discrimination tasks. Nevertheless, given the striking asymmetry observed in VEP modulation dynamics in the present experiment, it is difficult to imagine that attention was not exerted asymmetrically between the 2 visual fields. Notably, this is not the first demonstration of a specifically upper field reduction in C1 amplitude in the context of a visual texture discrimination task (Pourtois et al. 2008). In that case, the reduction was as a result of perceptual learning rather than spatial attention but the same arguments in favor of V2 involvement could still apply. Similarly to our upper field suppression effect, this effect also stands in contrast to reports of increased C1 amplitude by learning (Bao et al. 2010; Zhang et al. 2015), which were observed instead in the context of orientation judgment tasks. Thus, our unusual upper field suppressive effect of the C1 and its discrepancy with respect to other findings is strikingly mirrored in the perceptual learning literature where the discrepancy also aligns with the context of making textural versus nontextural judgments. This lends support to our suggestion that differential emphasis on texture detection is a viable explanation for the discrepancy in our case. It is therefore tempting to conclude that the divergent strategy between experiments 2 and 3 was that in experiment 3, V2 was selectively boosted in the upper visual field in order to better detect a textural target (a strategy that was missed by participants in experiment 2 due to the less optimal learning conditions). While this idea provides a unified explanation for a number of our results that would otherwise be difficult to link (C1 suppression by attention, C1 suppression under grating (as opposed to disk) target conditions, the early onset of the P1, the superior performance in experiment 3 compared with experiment 2), it is nevertheless quite a speculative account and purpose-built experiments need to be conducted in the future to address the question. The V2 account does not address the absence of a lower field P1 modulation for both target types in experiment 3 however. With this finding, it seems likely that attentional selectivity took place at a later latency for lower field stimuli in this experiment, although the reason for this is unclear. Thus, the full gamut of unexpected, asymmetric attention patterns in this experiment clearly call for further dedicated investigation to fully resolve them.

Whatever the neural underpinnings of the asymmetrical VEP effects in experiment 3, it seems quite likely that they correspond to an adaptive strategy that was suited to this particular target-detection task, which participants tended not to learn without the aid of performance feedback. Therefore, it is difficult to say how representative the results of this experiment are to the wider context of target-detection tasks. Thus, while this experiment does not support the core hypothesis that C1 modulation by spatial attention relies on feature-matching of the target and probe stimuli, it is difficult to draw a strong conclusion on this question based on this experiment. In fact, we would contend that this question remains very much open for a number of reasons. Comparison between experiments continues to suggest that C1 modulations do depend on target feature matching as the experiments that showed the modulation matched target features—experiments 1 and 2 here and Kelly et al. (2008)—while the experiments that did not show the modulation did not match target features in that low spatial frequency components that were present in the target were absent in the nontarget probes (Baumgartner et al. 2018; Alilović et al. 2019; see Introduction), and the disk-detection condition of experiment 3 here, with the only anomaly being the Gabor-detection condition of experiment 3 discussed above. Indeed, the very fact that C1 modulation dynamics were different between experiments 2 and 3 and between the Gabor and disk-detection tasks suggests that early visual responses are amenable to top–down control and that this top–down control can be exerted flexibly depending on the nature of the task. Nevertheless, a clear and direct demonstration that C1 modulation relies on feature similarity between the target and probe remains to be shown. Future research might compare, within subjects, the stimuli used in experiment 1 here and in Kelly et al. (2008) with those used by Baumgartner et al. (2018) and Alilović et al. (2019) to test this hypothesis further. It will also be incumbent for future research to explore alternative feature-matched targets to clarify how general this C1 modulation is to different target-detection contexts.

To end with a final point on methodology, by comparing CSD and non-CSD transformed waveforms, we were able to observe dynamics of the VEP that would not have been apparent from either waveform alone. Although we could not confidently infer the neural underpinnings of the observed differences in the absence of any independent verification, they nevertheless prompted inferences about the potential impact of signal overlap that may be helpful to generate hypotheses for future experiments. Although our inferences in this context relied heavily on prior knowledge about the structure of the early visual cortex, the practice may nevertheless prove useful in other contexts to expand the insights that can be drawn from EEG data.

## Conclusion

The question of whether spatial attention modulates the C1 component of the visual evoked potential has been controversial for a number of decades. With a large number of subjects (*N* = 45), we show that the C1 can be modulated by spatial attention, at least under some circumstances. Performance feedback did not affect the size of this effect and while the impact of target-probe feature similarity was not entirely clear, no modulation was observed when target and probe features were mismatched, as expected. We suggest that one factor that has brought difficulty to the detection of C1 modulations is that overlapping signals of opposite polarity, such as V1 and V2, may contribute to the component, each potentially exhibiting modulations of opposite polarity that cancel out on the scalp. Future research of the C1 component will benefit greatly from reliable methods of resolving distinct contributions from different sources. Another such factor is that the deployment of attention at this early processing stage may be flexible yet specific, making the observation of modulations particularly sensitive to the nature of the task. While the present paper has not decisively demonstrated this factor, it remains an exciting avenue for future C1 research. Indeed, the very fact that the C1 seems more sensitive to particular aspects of the experimental paradigm than are later components such as the P1 and N1, make it an ideal component to probe the nuanced strategies that the human brain employs in the direction of its attentional resources.

## Supplementary Material

Supplementary_tgaa045
