# Supplementary material for: Modulation of the Earliest Component of the Human VEP by Spatial Attention: An Investigation of Task Demands
Source: Cereb Cortex Commun. 2020 Aug 5;1(1):tgaa045. doi: 10.1093/texcom/tgaa045 (PMC8152881; doi:10.1093/texcom/tgaa045)
Supplement: Supplementary_tgaa045 [file supplementary_tgaa045.docx]

Supplementary Table 1. Results of statistical tests are shown across a range of latency windows for measurement of C1 amplitudes. Furthermore, results pertaining to the impact of individually chosen latency ranges and common latency ranges are shown.

**
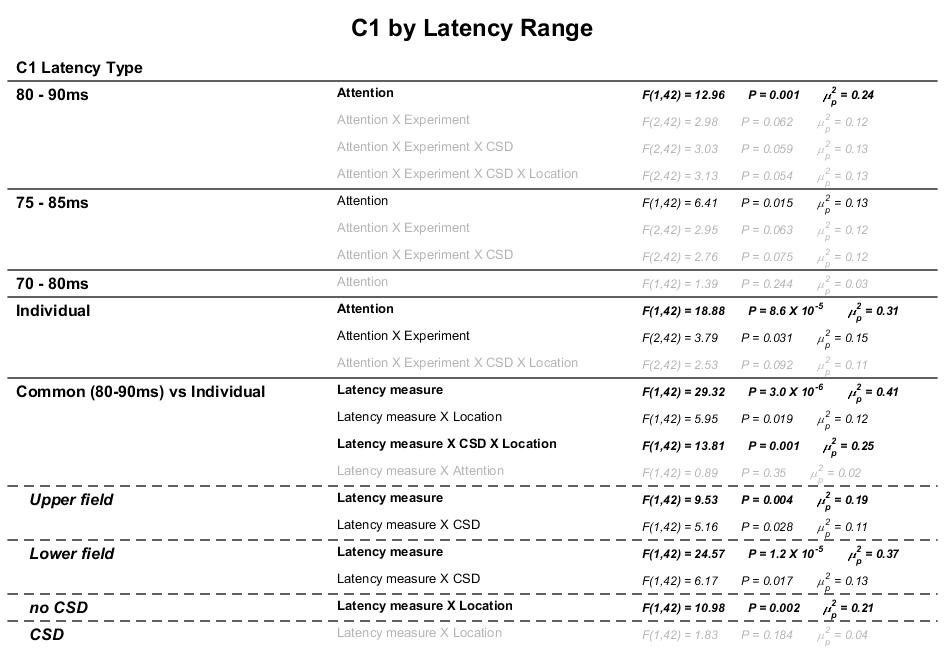
**


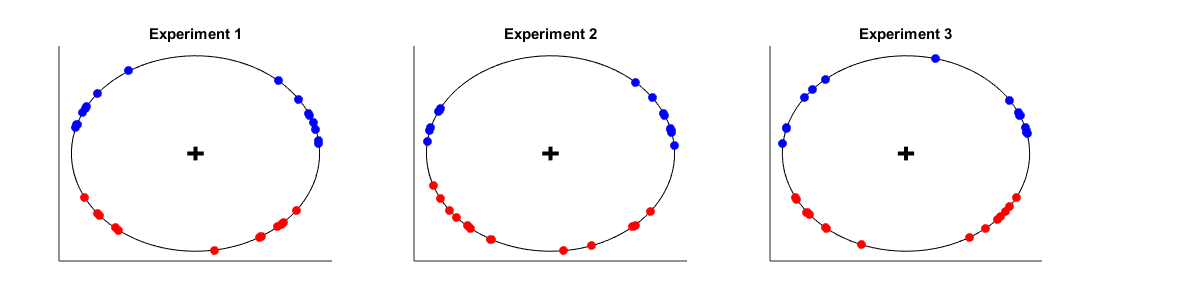


Supplementary figure 1. Polar coordinates of the chosen stimulus locations for each participant across all three experiments. Lower-field coordinates are shown in red while upper-field coordinates are shown in blue.


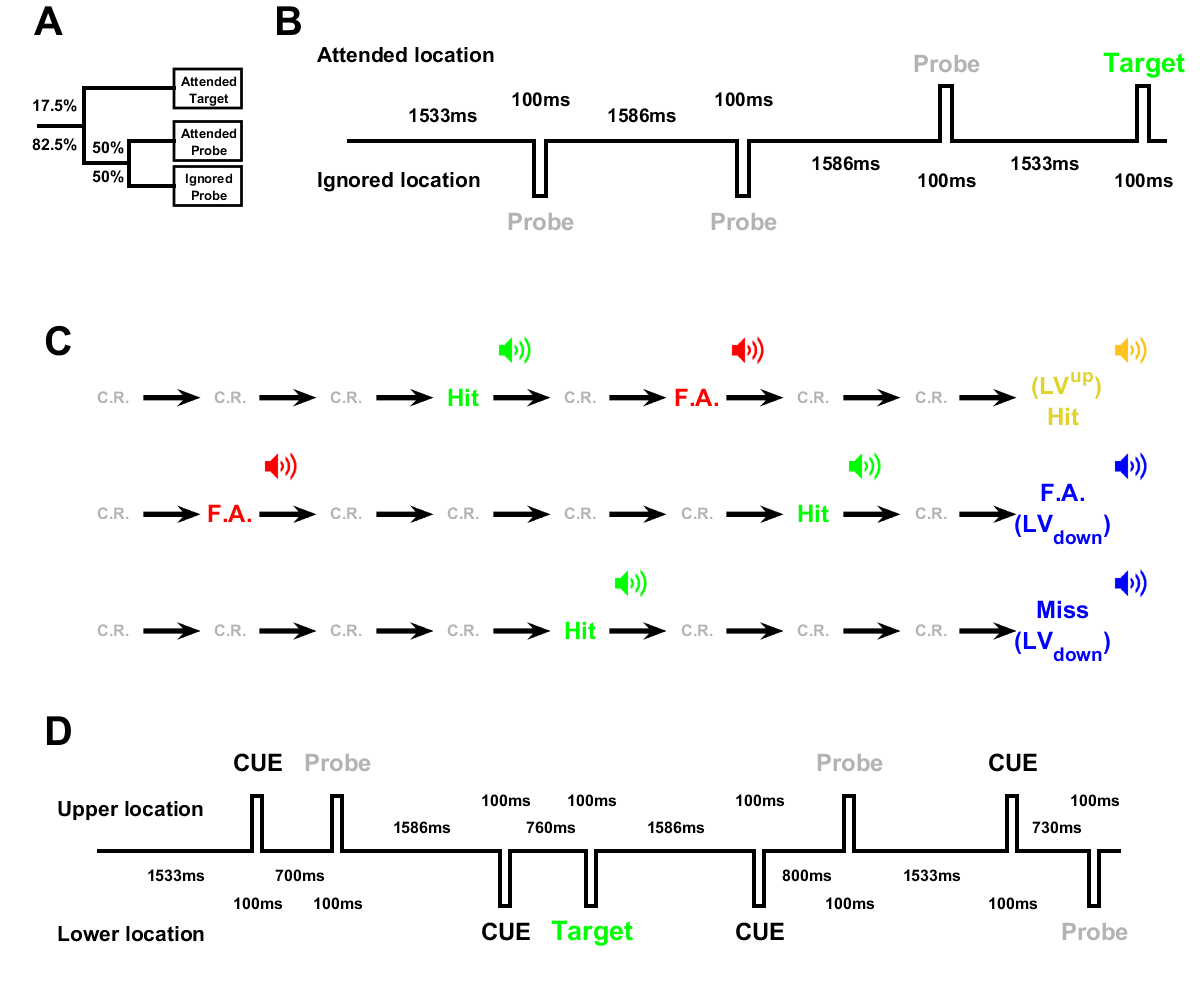


Supplementary figure 2. **A)** Tree diagram of the distribution of trial conditions within each block; the attended location cycles between lower and upper twice in each block. **B)** An example of four consecutive trials in experiments 1 and 2, showing the stimulus types and durations, the inter-stimulus-intervals, and the stimulus locations (represented by the direction of deflection). **C)** Three examples of a 9 consecutive trial sequence are shown. The abbreviations are false alarm (F.A.), LV (level) and correct rejection (c.r.). The colours indicate the online feedback with grey indicating no feedback, green indicating a “first hit” tone, red indicating a “first false alarm” tone, gold indicating a levelling up tone along with an on-screen print of the new level, and blue indicating a levelling down tone along with an on-screen print of the new level. **D)** An example of four consecutive trials in experiment 3, showing the additional trial by trial cue and cue-to-stimulus interval.


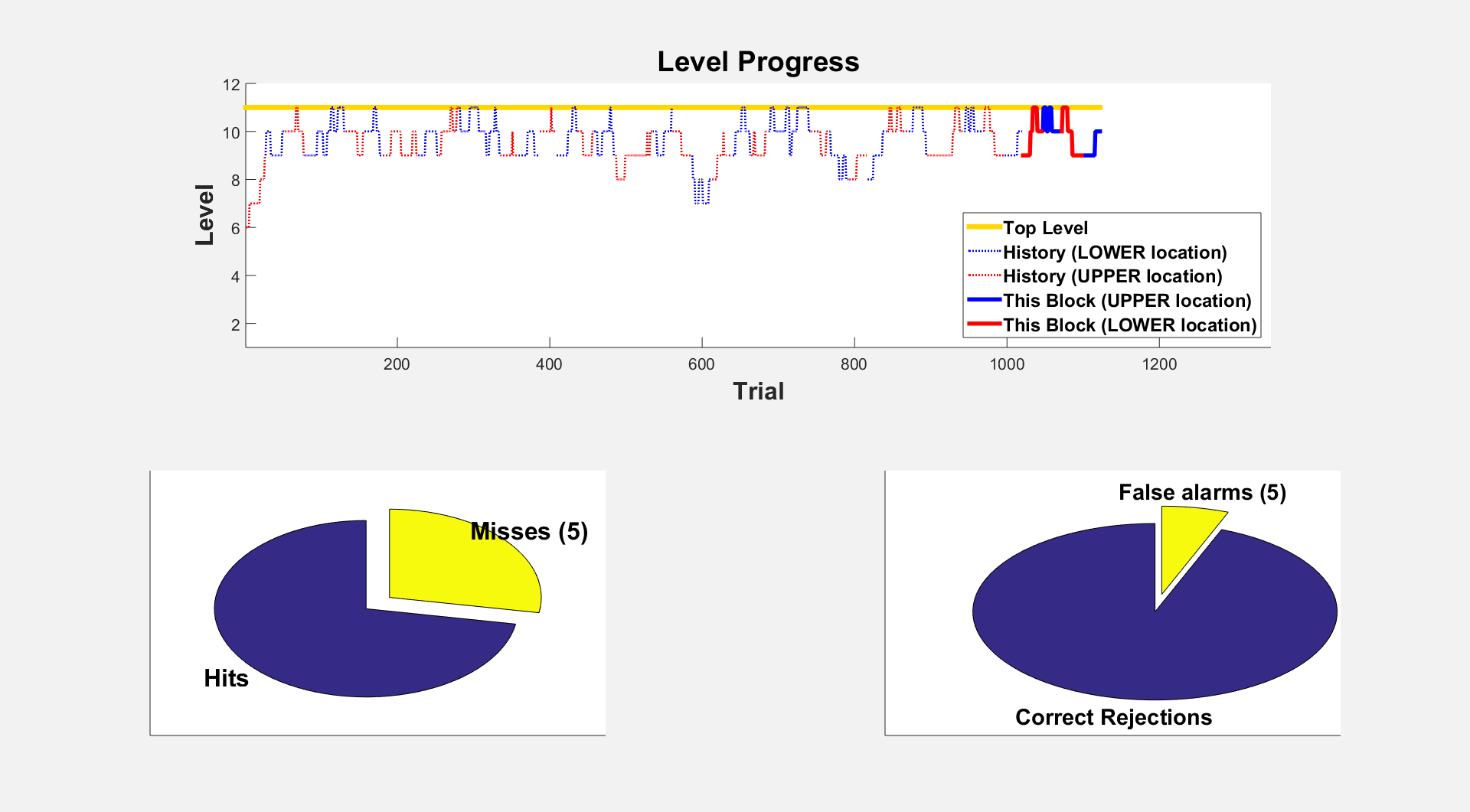


Supplementary figure 3. An example of the end-of-block feedback received by participants in experiments 1 and 3 and the motivated condition of experiment 2. On the top, blue and red line segments show difficulty levels when the upper and lower locations were attended respectively, with the current block shown in continuous line and previous blocks shown in dashed line. On the bottom, pie charts show the number and proportion of misses and false alarms in the present block only.


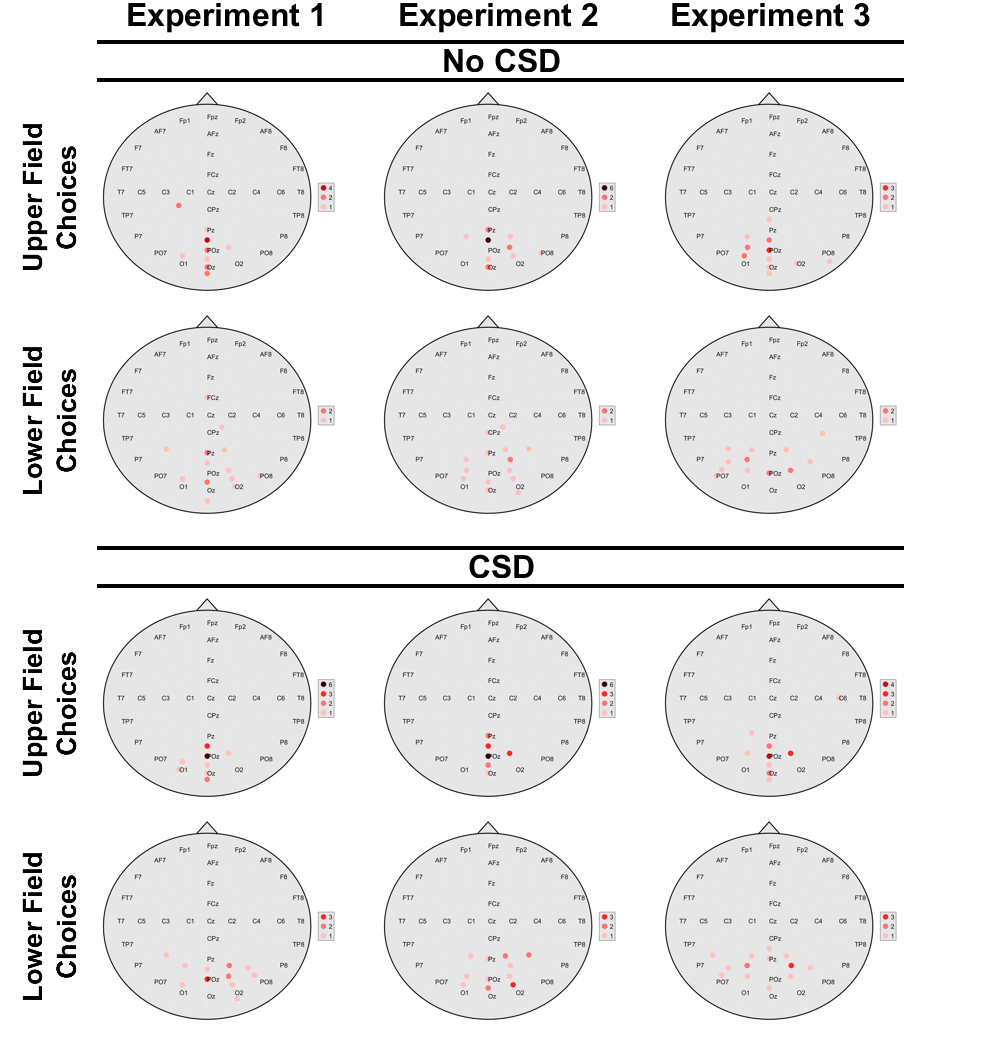


Supplementary figure 4. Scalp map of electrode choices for C1 measurement. Electrode choices for C1 measurement in experiment 1 (left), experiment 2 (middle) and experiment 3 (right) are shown for non-CSD (top) and CSD (bottom) transformed data. Electrode choices are shown relative to the 10-20 electrode sites that are included in the Biosemi ABC layout.


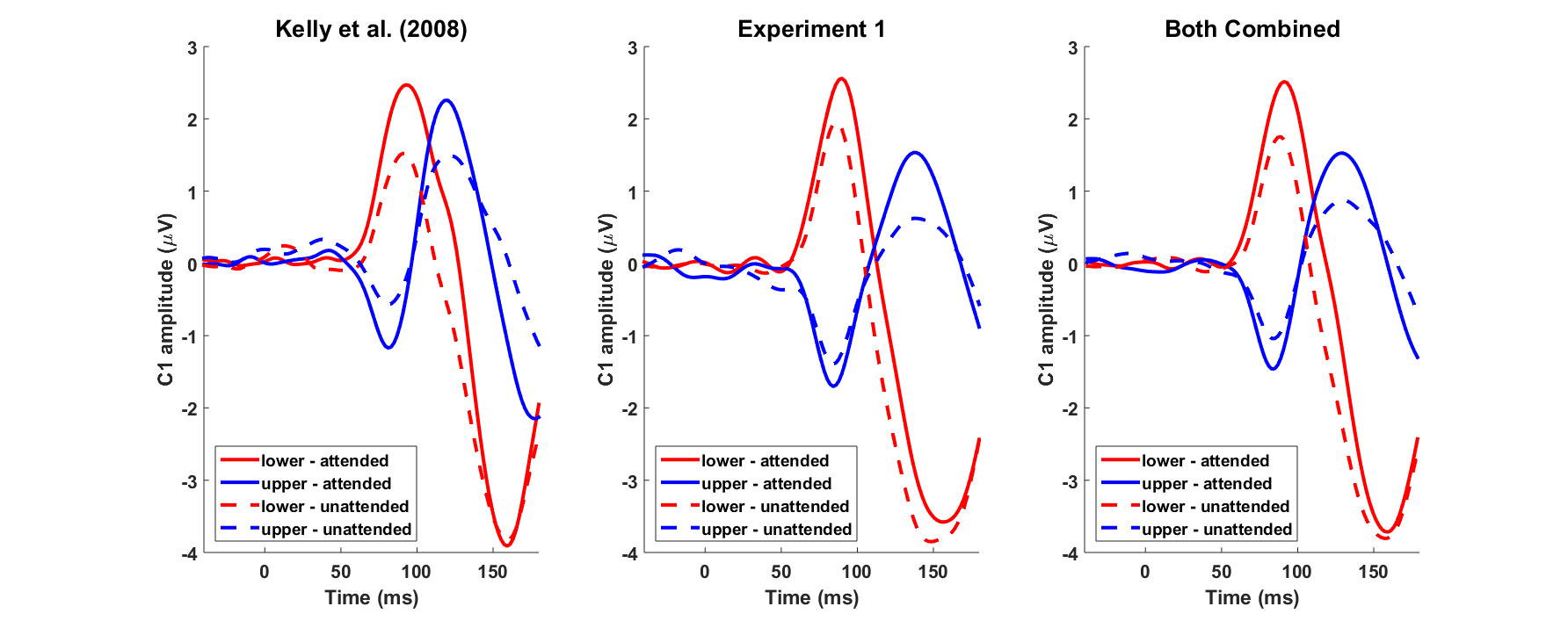


Supplementary figure 5. Waveforms demonstrating C1 modulation in the original study by Kelly et al. (2008) (left), the present experiment 1 (middle), and with participants from both experiments combined (right). Note that in order to conform to the analysis protocol of the original study, these waveforms are baseline corrected to the range -80 to 0 ms rather than -50 to 30 ms.


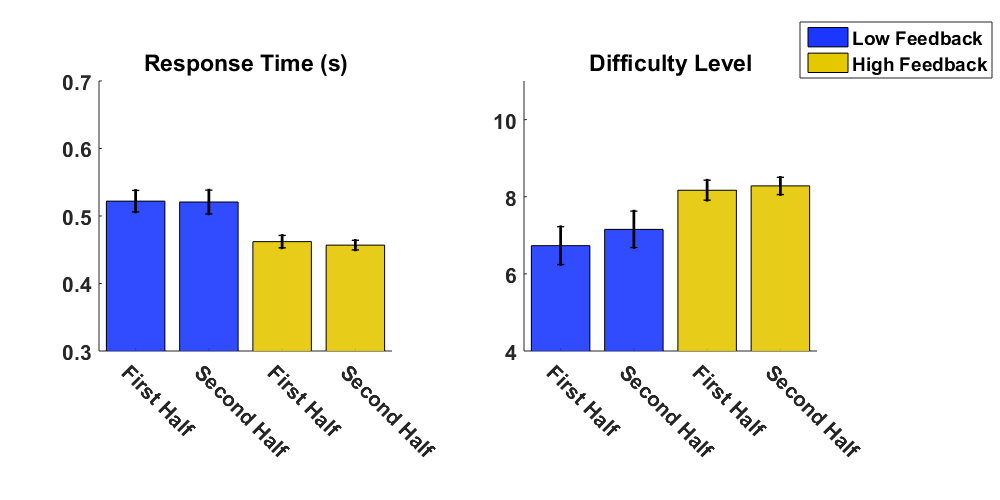


Supplementary figure 6. Response time and average difficulty level across each quarter of experiment 2. The bar plots are colour-coded according to feedback condition but since the Low Feedback condition was always done first, the two halves of that condition correspond to the first two quarters of the experiment.


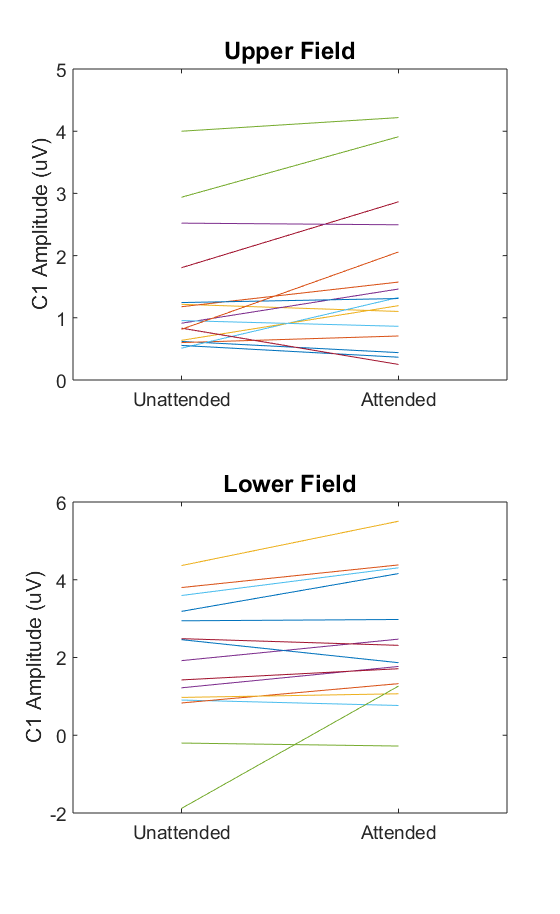


Supplementary figure 7. Individual C1 amplitudes in the Ring-target experiment (Experiment 1) for non-CSD transformed data (using the 80-90 ms C1 measurement window).


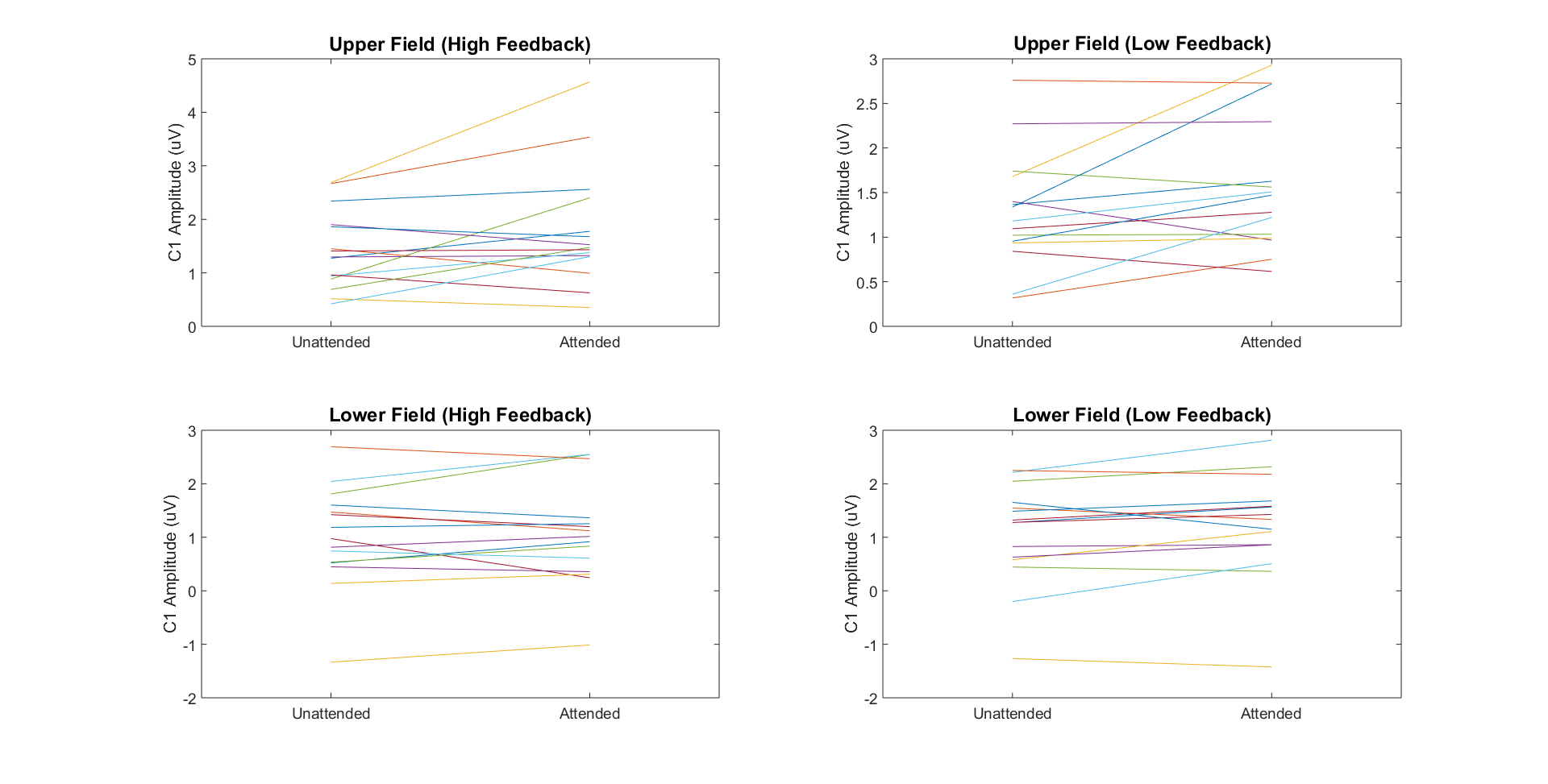


**Supplementary figure 8. Individual C1 amplitudes in the Feedback experiment (Experiment 2) for non-CSD transformed data.**


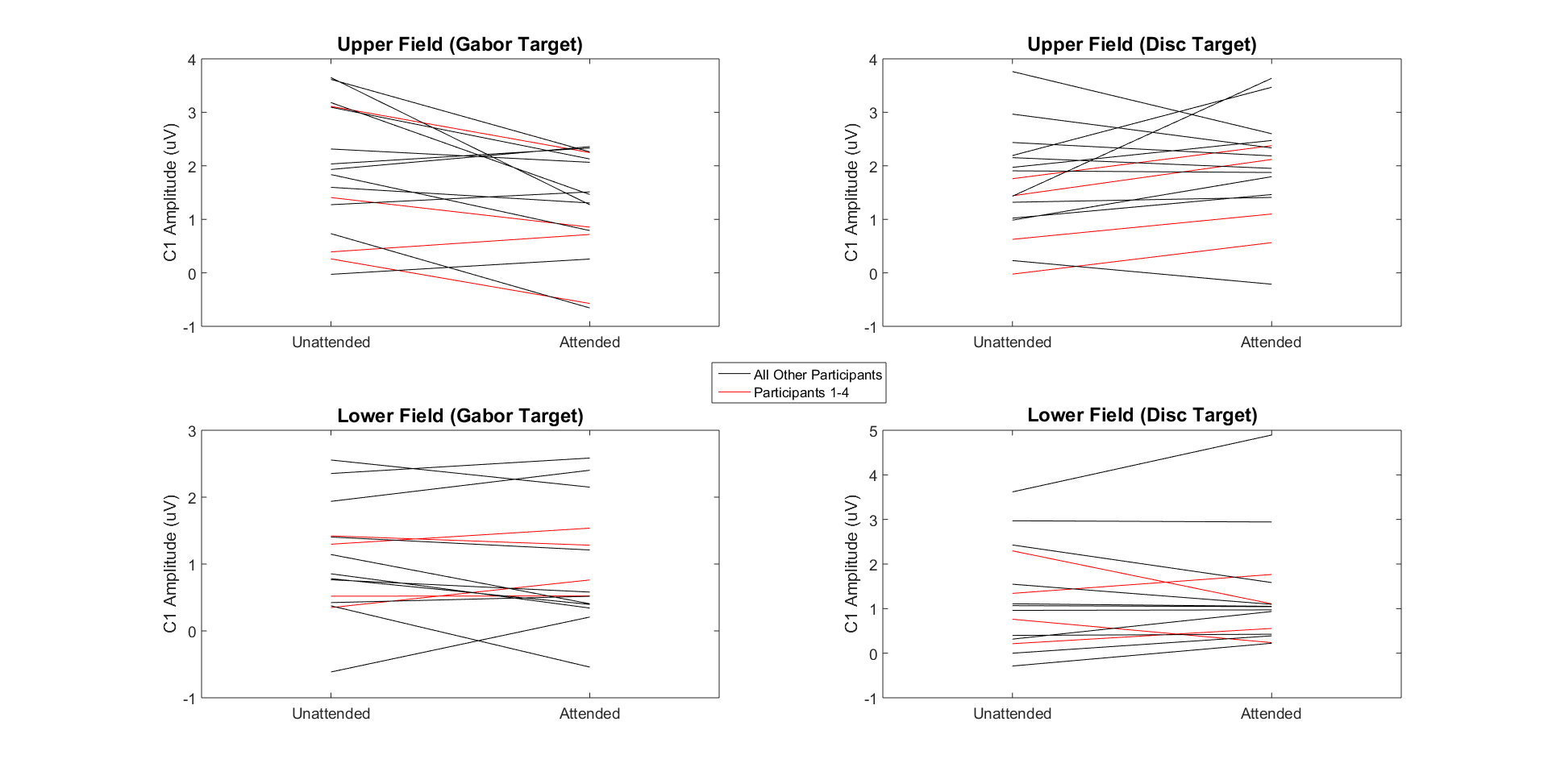


Supplementary figure 9. Individual C1 amplitudes in the Target-type experiment (Experiment 3) for non-CSD transformed data.


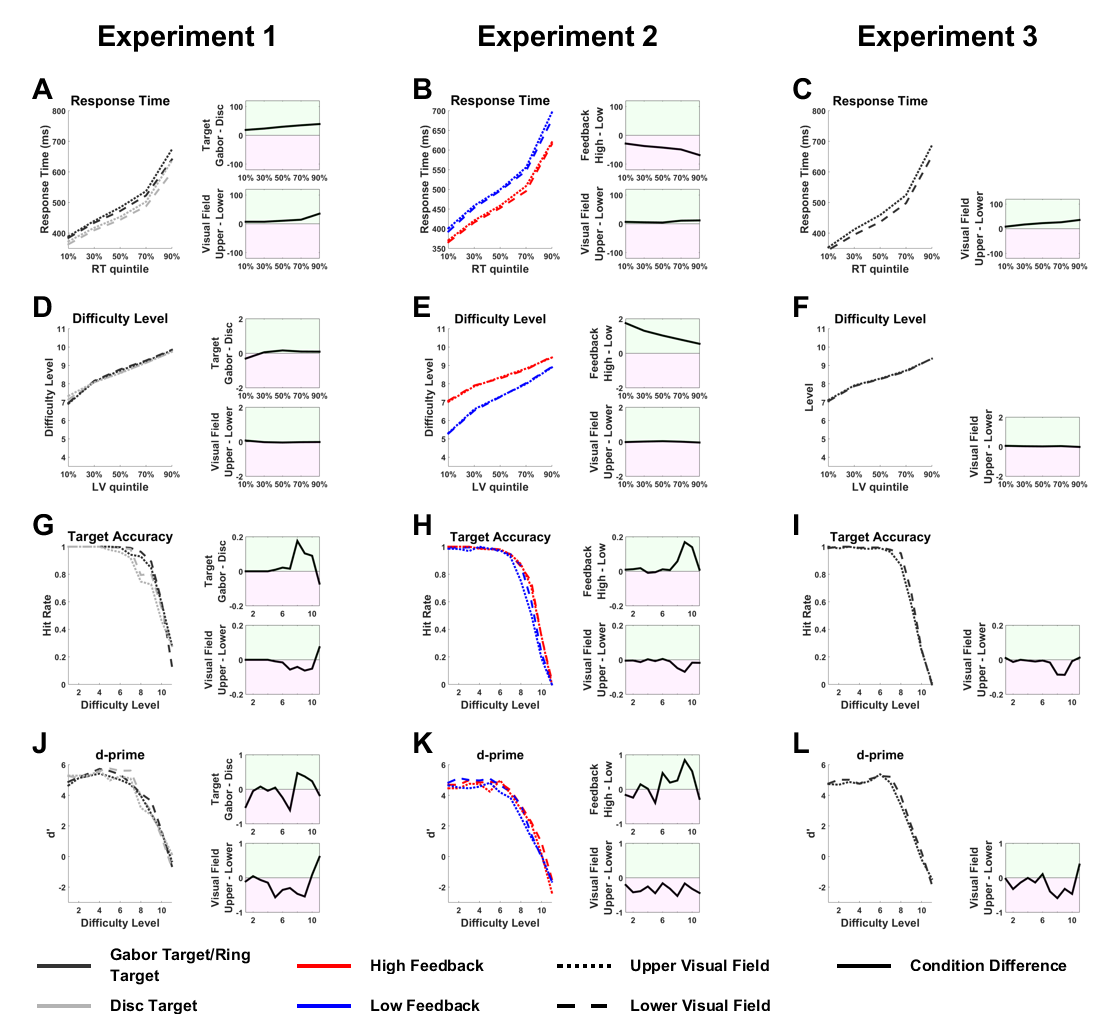


Figure 10. Behaviour across all three experiments. Response times (top row), difficulty level achieved (second row), target accuracy/hit rate (third row) and d’ (last row) are depicted for each experiment (columns), broken down by target type (experiment 1), feedback level (experiment 2), and visual field location (all experiments). Response times and difficulty level achieved are divided into quintile bins while target accuracy and d’ are divided into bins based on difficulty level. Adjacent to each figure are difference plots showing the difference between target conditions (experiment 1) or feedback level (experiment 2) on the top, and the difference between visual field location (all experiments) on the bottom.


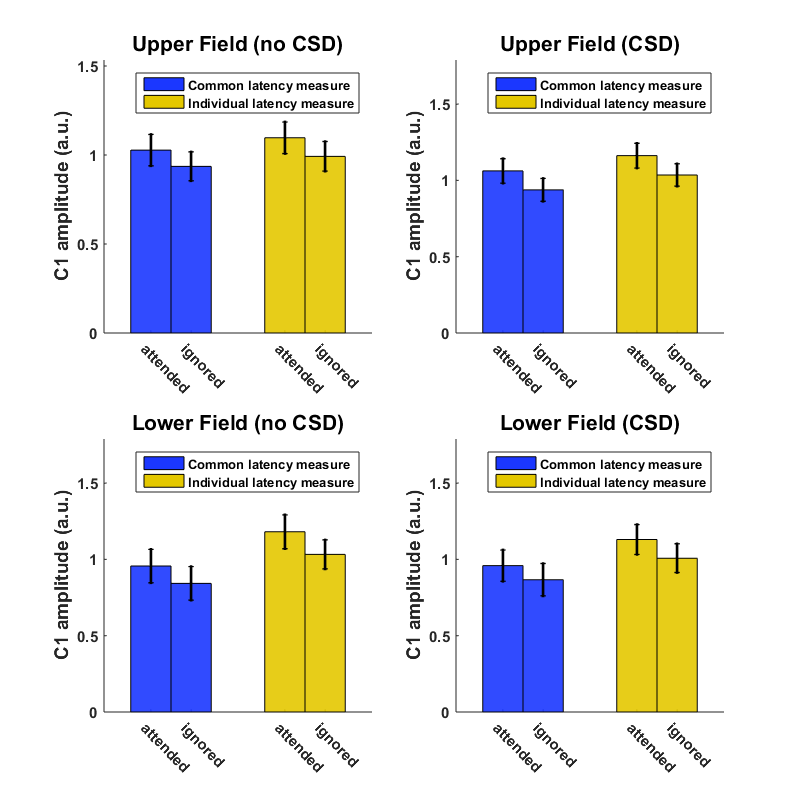


Supplementary figure 11. C1 amplitudes as a function of C1 latency measure. Bar charts depict normalized C1 amplitudes, comparing the common latency measure of 80-90 ms to individual participant latency windows for non-CSD (left) and CSD (right) transformed data, and for the upper (top) and lower (bottom) visual field.
